# The Chromosome-Level Genome of *Hestina assimilis* (Lepidoptera: Nymphalidae) Reveals the Evolution of Saprophagy-Related Genes in Brush-Footed Butterflies

**DOI:** 10.3390/ijms24032087

**Published:** 2023-01-20

**Authors:** Lu Zhao, Xiao-Dong Li, Tao Jiang, Hang Wang, Zhicuo Dan, Sheng-Quan Xu, De-Long Guan

**Affiliations:** 1 College of Life Sciences, Shaanxi Normal University, Xi’an 710119, China; 2 School of Chemistry and Bioengineering, Hechi University, Yizhou 546300, China

**Keywords:** chromosome-level genome, comparative genomics, *Hestina assimilis*, CYP450, chemoreception, saprophagy

## Abstract

Most butterflies feed on nectar, while some saprophagous butterflies forage on various non-nectar foods. To date, little is known about the genomic and molecular shifts associated with the evolution of the saprophagous feeding strategy. Here, we assembled the high-quality chromosome-level genome of *Hestina assimilis* to explore its saprophagous molecular and genetic mechanisms. This chromosome-level genome of *H*. *assimilis* is 412.82 Mb, with a scaffold N50 of 15.70 Mb. In total, 98.11% of contigs were anchored to 30 chromosomes. Compared with *H. assimilis* and other Nymphalidae butterflies, the genes of metabolism and detoxification experienced expansions. We annotated 80 cytochrome P450 (CYP) genes in the *H. assimilis* genome, among which genes belonging to the CYP4 subfamily were significantly expanded (*p* < 0.01). These P450 genes were unevenly distributed and mainly concentrated on chromosomes 6–9. We identified 33 olfactory receptor (OR), 20 odorant-binding protein (OBP), and six gustatory receptor (GR) genes in the *H. assimilis* genome, which were fewer than in the nectarivorous *Danaus plexippus*. A decreased number of OBP, OR, and GR genes implied that *H. assimilis* should resort less to olfaction and gustation than their nectarivorous counterparts, which need highly specialized olfactory and gustatory functions. Moreover, we found one site under positive selection occurred in residue 996 (phenylalanine) of GR genes exclusive to *H. assimilis*, which is conservative in most lineages. Our study provides support for the adaptive evolution of feeding habits in butterflies.

## 1. Introduction

Evolutionary shifts related to changes in feeding habits are considered one of the most critical events in animal evolution [1,2,3]. The brush-footed butterfly (family Nymphalidae) is one of the most species-rich butterfly taxa, with more than 6000 species [4]. While most adults are nectarivorous flower visitors and strongly depend on flower nectar and pollen, some butterflies forage for various non-nectar foods, such as rotting fruit, honeydew, tree sap, mud, carrion, and dung [5,6,7]. These species have significant morphological variation in their proboscis, which appears to be related to their specialization and adaptation concerning the physical properties of humus [8] and physiological characteristics associated with the processing of non-nectar foods. Non-nectar foods differ significantly from flower nectar in their chemical properties, including low sugar concentrations and fermentation products (ethanol and acetic acid) [5,9]. Research shows that ethanol and acetic acid, together with low concentrations of sugar, synergistically stimulate the feeding behavior of saprophagous butterflies [5,10]. Although such feeding habits are intriguing from the ecological and evolutionary aspects of food utilization in adult butterflies, the genomic and molecular shifts associated with the evolution of the saprophagous feeding strategy remain largely unknown.

*Hestina assimilis* Linnaeus (1758) is a larger species of Nymphalidae with strong adaptability and a wide distribution [11]. It recognized by 4–5 red spots on the outer edge of its hind wing; thus, it is commonly known as a red ring skirt [12]. It is a typical saprophagous butterfly, and the adults can utilize the diluted liquid of rotting foods such as tree sap, rotting fruits, and even feces. Due to this trait, the survival range and population size of *H. assimilis* can expand continuously [13]. Therefore, *H. assimilis* can serve as an ideal model for exploring the mechanisms of saprophagy [14].

Cytochrome P450 monooxygenases (CYPs) can specifically and non-specifically neutralize various types of humus [15,16,17]. Most CYP3 genes are involved in the metabolic detoxification of secondary plant substances to help insects better adapt to host plants [18,19]. The CYP4 subfamily has been associated with lipid metabolism, epidermal hydrocarbon formation, and insect olfactory recognition regulation [20]. Several CYP2 and mitochondria P450 genes play vital roles in regulating the growth and development or preventing the invasion of pathogens and substances [20,21]. Chemoreceptions provide attractive models to understand how organisms adapt to diverse diets because they lie between external environmental signals and internal physiological responses [22]. Olfactory receptors (ORs) and odorant-binding proteins (OBPs) participate in the olfactory response of insects by recognizing and binding host plant volatiles [20]. Olfactory responses are fundamental to the life cycles of insects, which they use for foraging, communicating with conspecifics, and recognizing predators [23]. The differences in food selection are likely to reflect the difference in their gustatory responsiveness. Gustatory receptors (GRs) are well known for their functions in sensory neurons in detecting food and toxins [24]. The expression of GRs across different tissues and sexes is a strong candidate for mediating unusual feeding behavior [25]. Therefore, identifying these gene families is beneficial for elucidating the mechanism of the dietary transition in immunity, recognition, and detection.

Here, we generated a chromosome-level reference genome of *H. assimilis*, and used a comparative genomic approach to identify gene families that could facilitate saprophagous behavior or physiological means. This work serves as a valuable genomic resource for understanding the ecological and evolutionary characteristics of *H. assimilis*.

## 2. Results

### 2.1. High-Quality Chromosome-Level Assembly, Gene Prediction, and Functional Annotation

We obtained 62.39 Gb (147×) of nanopore long reads and 69.43 Gb (165×) of Illumina paired-end reads to construct the contig-level genome for this butterfly. We yielded a 422.61 Mb assembly (scaffold number: 53, N50: 14.28 Mb), which was consistent with the genome size estimated based on the k-mer analysis (421.41 Mb) (Figure S1). Combined with the 88.21 Gb (200×) of Hi-C fragment libraries sequencing, 98.11% of scaffolds from *H. assimilis* anchored onto 30 chromosomes, ranging from 5.17 Mb to 23.25 Mb in length (Figure S2), which suggested that the karyotype of *H. assimilis* was 30. Finally, a 412.82 Mb assembly (scaffold N50: 15.70 Mb; GC content: 35.45%) of haplotype genomes without a heterozygous sequence was yielded with 95.80% completeness of the BUSCO analysis (lepidoptera_odb10 database), which was adequate for further research.

We identified ~35.07% of the assembled genomes as repetitive sequences (Table S1). Among them, the repetitive sequences dominated with long interspersed nuclear elements (LINEs, 9.56%) and short interspersed nuclear elements (SINEs, 9.53%). We compared the repeats of *H. assimilis* with other species in the same family, and the proportions and length of repeats in each species were not correlated with dietary differences (Figure S3). After masking the repeat elements, we identified 16,120 protein-coding genes with a 95.10% BUSCO value in the *H. assimilis* genome. In total, 12,095 (75.03%) and 13,965 (86.63%) predicted genes were supported through the use of functional annotation from the InterProScan and NR databases (Tables S2 and S3). The average lengths of genes and CDSs were 9983 bp and 1440 bp, respectively, compared to other Lepidoptera species (Figure S4).

### 2.2. Synteny Analysis

**Figure 1:**
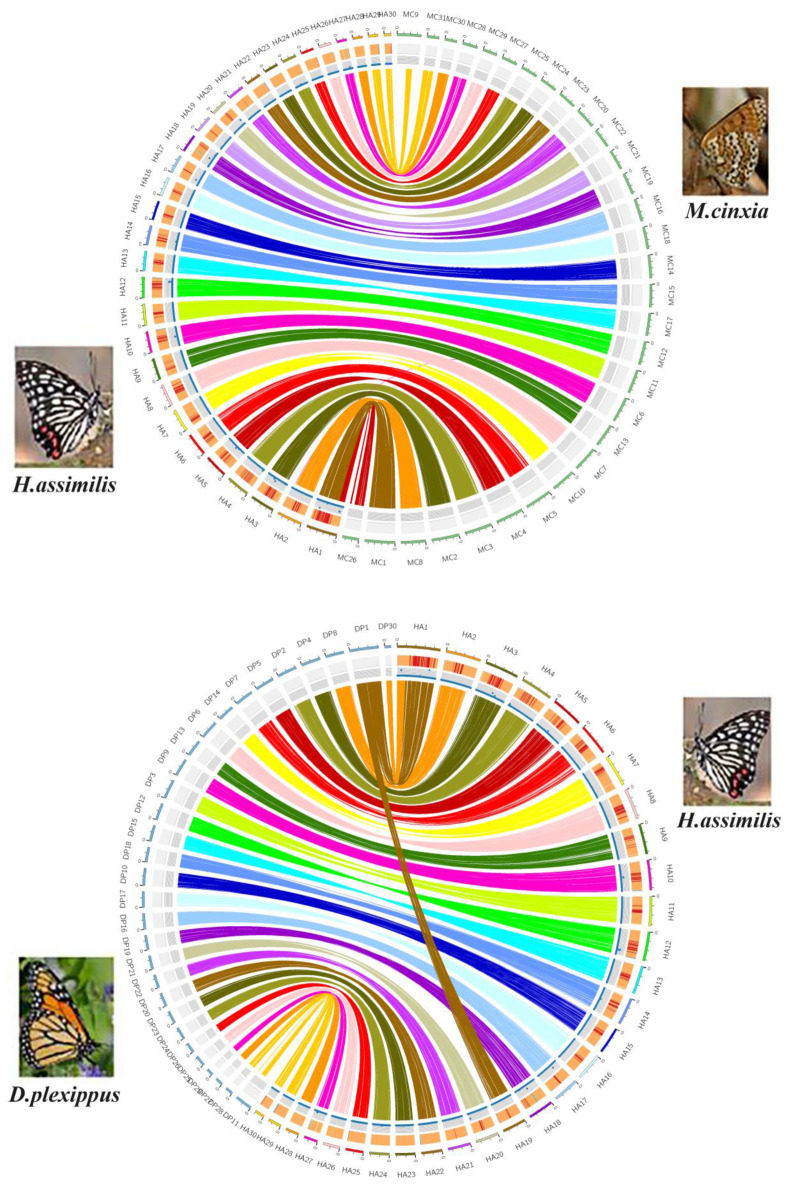
Synteny alignment of the genome of *H. assimilis* (male), *M. cinxia* (male), and *D. plexippus* (male). The tagged HA01 to HA30 refer to the chromosomes of the sequenced *H. assimilis*, the green columns refer to the chromosomes of *M. cinxia*, and the blue columns refer to the chromosomes of *D. plexippus*. HA represents the *H. assimilis* chromosome; MC represents the *M. cinxia* chromosome; DP represents the *D. plexippus* chromosome. The HA01 is syntenic to the MCZ chromosome and autosomes MC26 of *M. cinxia* and the DPZ chromosome and autosomes DP30 of *D. plexippus*. The densities of GC contents (34% to 36%) and DNA variations (0% to 0.3%) in *H. assimilis* are shown in the outer rims in order.

Using *Melitaea cinxia* as a reference, a synteny plot revealed the sex chromosomes among 30 super-scaffolds. Our results showed that chromosome one of *H. assimilis* was syntenic to the Z chromosome (chromosome one) and autosomes (chromosome 26) of *M. cinxia* (Figure 1). Then, we assessed the sequencing coverage and constructed 3D chromatin structure analyses using an Hi-C read pair. The average coverage was consistent across the entire Z chromosome length. Moreover, the frequency of the Hi-C read pair interactions was the same as that of other unfused genomic regions, suggesting a Z-autosome fusion event in *H. assimilis* (Figure S5). To further confirm this event, we performed the homology-based chromosomal assignments using *Danaus plexippus* as a reference, concluding that the Z-autosome fusion did exist in *H. assimilis* (Figure 1).

### 2.3. Phylogenetic and Positive Selection Analysis

**Figure 2:**
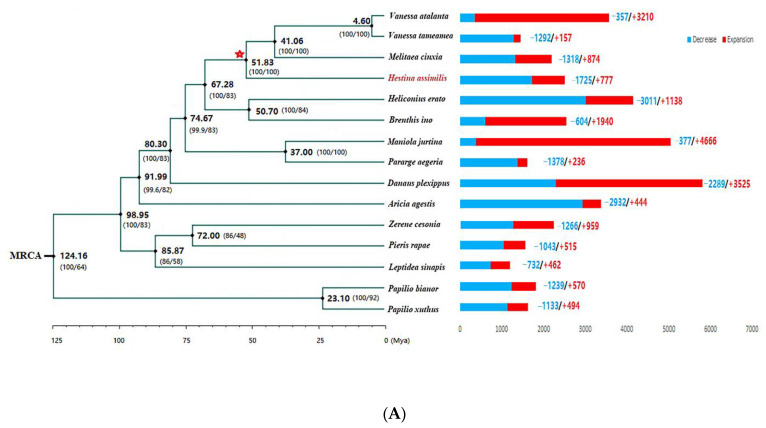

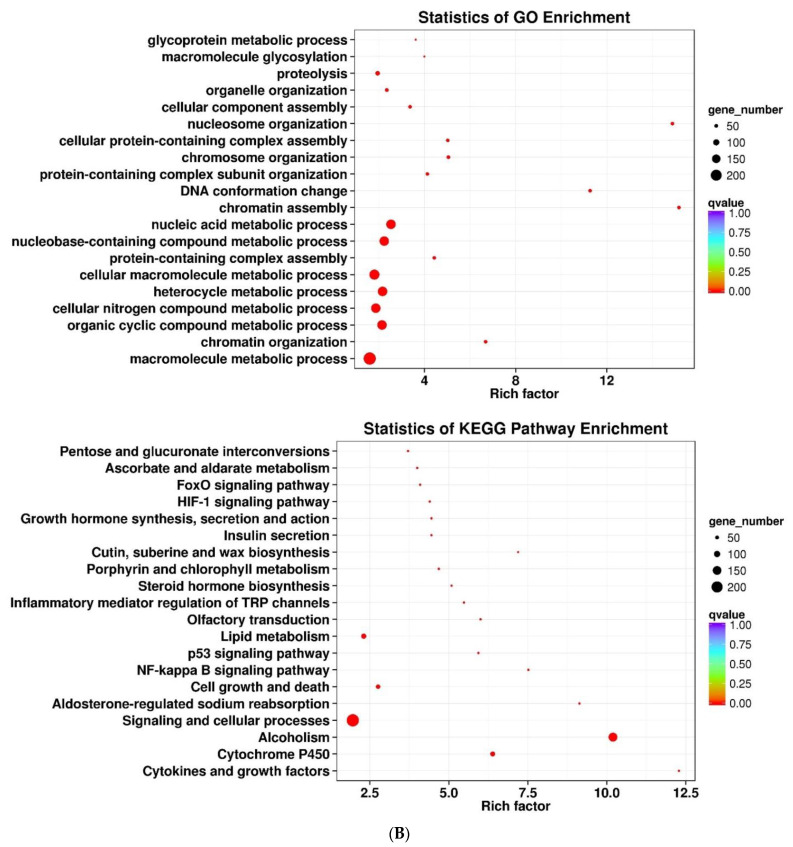
Phylogenetic and gene family analysis of *H. assimilis.* (**A**) Time-calibrated phylogeny of the fifteen butterfly species based on single-copy orthologous genes. Numbers on the bar charts indicate the numbers of gene families that experienced expansion (red) or contraction (blue); the number beside each node denotes the estimated divergence time (millions of years ago). Numbers in parentheses are SH-aLRT support (%)/ultrafast bootstrap support (%). The red star represents the common nodes of the cluster leading to four saprophagous species (*V. tameamea*, *V. atalanta*, *M. cinxia*, and *H. assimilis*). (**B**) GO and KEGG enrichment analysis of expanded gene families was visualized as a scatter plot. The vertical axis represents the path name, and the horizontal axis represents the gene ratio. The bubble size indicates the gene number, and the color corresponds to different q-value ranges.

We aligned the 1453 single-copy orthologous genes of the 13 species described (*Papilio bianor* and *Papilio xuthus* as the outgroup) to reconstruct a maximum-likelihood phylogenetic tree. The time-calibrated tree showed that the divergence time of *H. assimilis* was approximately ~51.83 Mya (Figure 2A).

We identified 14 positive selection genes specific to *H. assimilis*, including genes encoding bi-functional peptidase and arginyl-hydroxylase, delta-1-pyrroline-5-carboxylate dehydrogenase (P5CDh), B-cell receptor-associated protein, and the peroxisomal membrane protein (Table S4). These genes contribute to the energy supply and innate immunity to resist bacterial infection.

### 2.4. Gene Family Associated with Diet Metabolism Analysis

Regarding the gene families of 15 species to assign, *H. assimilis* had 777 expansion and 1725 contraction gene families (Figure 2A). In total, 117 significantly expanded (adjusted *p*-value < 0.01) gene families enriched biological process terms, including the macromolecule metabolic process (GO:0043170), organic cyclic compound metabolic process (GO:1901360), cellular nitrogen compound metabolic process (GO:0034641), and heterocycle metabolic process (GO:0046483) (Figure 2B, Table S5). KEGG pathways of the expanded gene families mainly included signaling and cellular processes, alcoholism, P450, and lipid metabolism (Figure 2B). Interestingly, the main metabolic signaling pathways, NOD-like receptor, AMPK, FoxO, and PI3K-Akt, were also expanded in the genome of *H. assimilis* (Table S6).

On the common nodes of the cluster leading to four saprophagous species (*Vanessa tameamea*, *Vanessa atalanta*, *M. cinxia*, and *H. assimilis*) (at the red star in Figure 2A)we predicted 87 expanded and 685 contracted gene families. Among them, 23 significantly expanded gene families enriched several GO categories, including the regulation of alcohol metabolism, nitrogen, heterocycle, and the aromatic compound catabolic process (Table S7).

### 2.5. Evolution of P450, OBP, OR, and GR Gene Families Related to Saprophagy in H. assimilis

**Figure 3:**
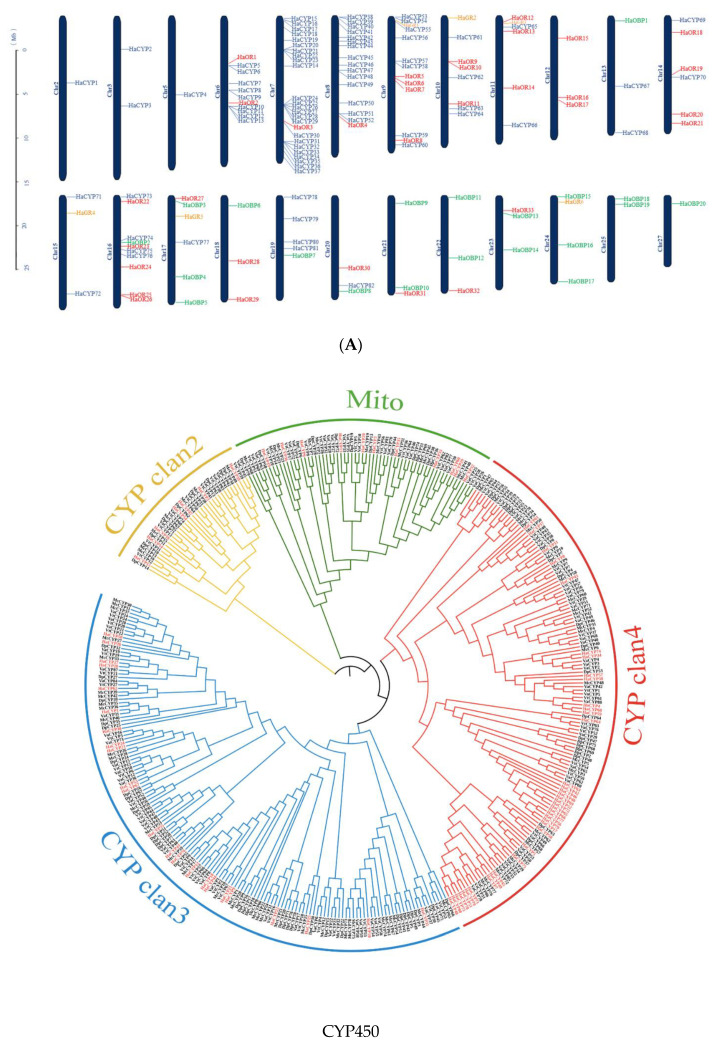

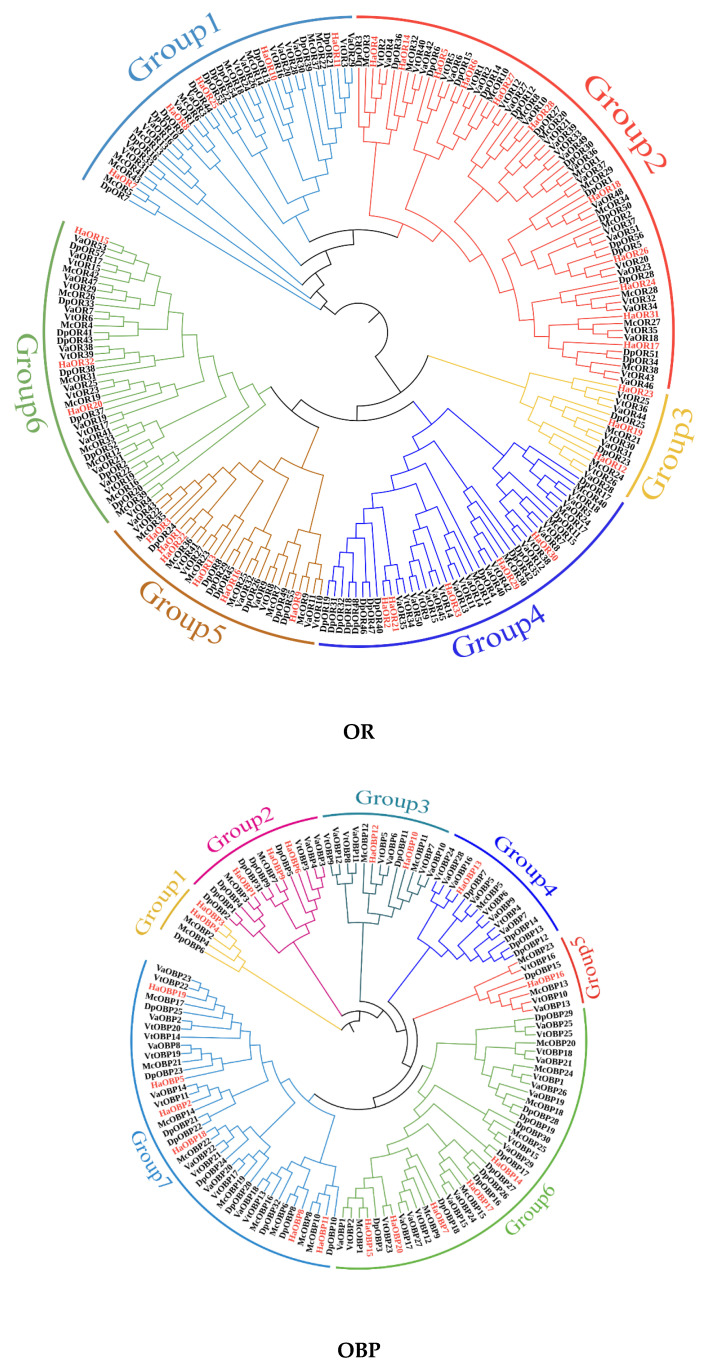

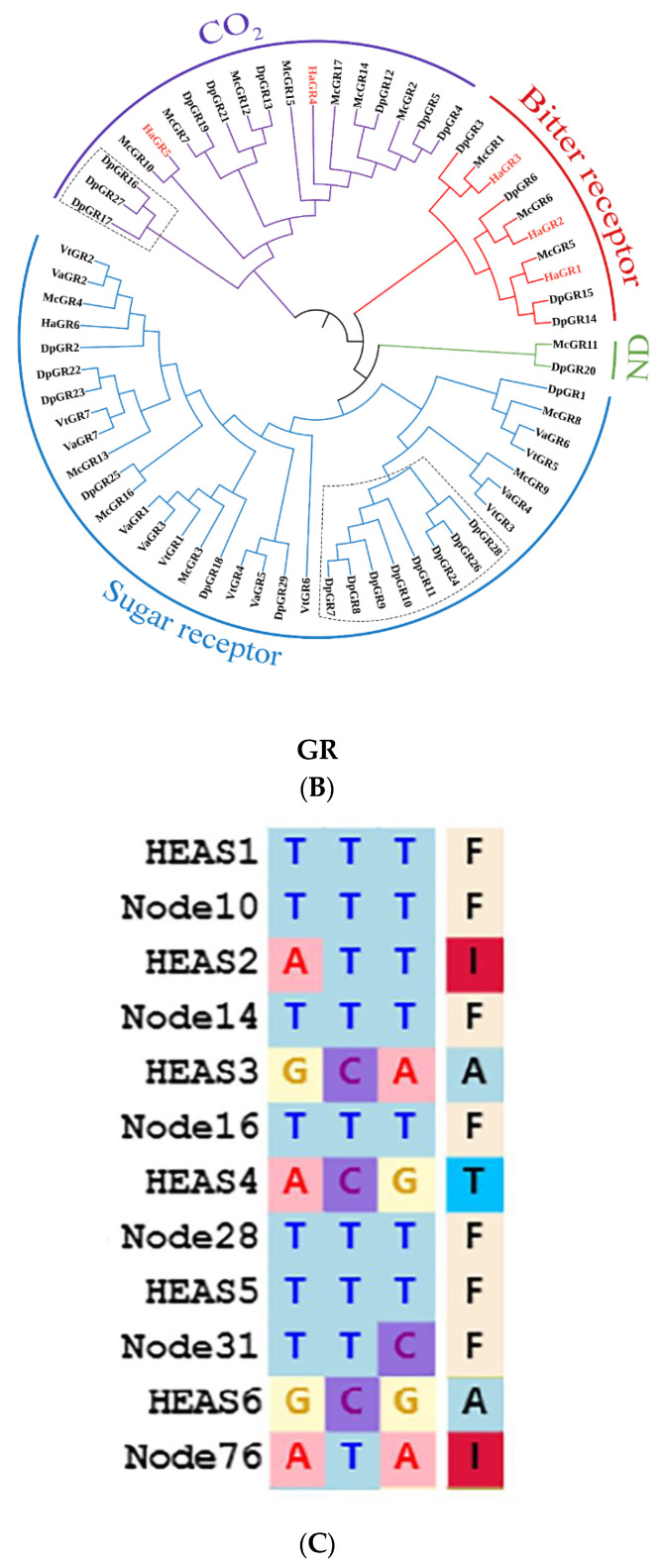
Chromosomal location and phylogenetic relationship of the detoxification and chemoreception gene families in *H. assimilis*. (**A**) Chromosome distribution of cytochromes P450 (CYP, blue), olfactory receptor (OR, red), odorant binding protein (OBP, green), and gustatory receptor (GR, yellow) genes. Chr2–27 represent chromosome numbers 02–27. (**B**) CYP, OR, OBP, and GR gene families in the genomes of *H. assimilis* (red), *M. cinxia*, *V. tameamea*, *V. atalanta*, and *D. plexippus*. (**C**) Non-synonymous substitution (996) in the CDS region of GR was significantly subject to positive selection.

We predicted 80 cytochrome P450s, 17 glutathione S-transferase, and 26 ATP-binding cassette transporters in *H. assimilis* (Table S8). These associated families also showed a pattern of general expansion in *H. assimilis*. We focused on the P450s, since their expansion was greatest in *H. assimilis*. The 80 CYP genes were unevenly distributed on the 16 chromosomes, and concentrated in chromosomes 6–9 (Figure 3A). The phylogenetic analysis found the 80 CYP genes divided into 18 families (Figure 3B). The phylogenetic relationships and chromosomal locations of the CYP genes revealed that the CYPs had independent gene duplication events in different chromosomal regions in *H. assimilis*. Compared with the four genomes of the Nymphalidae species, saprophagous feeders (*M. cinxia*, *V. tameamea,* and *V. atalanta*), and nectar feeders (*D. plexippus*), the number of CYP genes identified in *H. assimilis* was comparable to the other species. The P450 genes clustered with the lineages of CYP4 (39/80 genes), CYP3 (23/80 genes), CYP2 (11/80 genes), and the mitochondrial P450 clade (7/80 genes) (Figure 3B, Table 1). The expansions of the CYP4 and CYP3 clans in *H. assimilis* indicated its higher probability of encountering xenobiotics in living environments.

We found that the chemoreception gene family was contracted in *H. assimilis*. Then, we manually annotated 33 ORs, 20 OBPs, and 6 GRs (Table S9). We visualized the chromosomal distribution of these gene families. The ORs were distributed in the 15 chromosomes, and most OR genes were located on chromosome 16, with five ORs. OBPs were distributed across the 12 chromosomes, while six GRs were independently distributed on 6 chromosomes (Figure 3A). The phylogenetic tree indicated many deletions of chemoreception genes, explaining why *H. assimilis* have very different feeding habits. The genes in each cluster shared a common ancestor, as seen in the saprophagous *H. assimilis*, *M. cinxia*, *V. tameamea*, and *V. atalanta*, where these genes were distributed almost as singletons. The ORs and OBPs were divided into six and seven clusters: groups 1–6 and groups 1–7 (Figure 3B). Compared to other species, *H. assimilis* possessed the fewest OR and OBP genes, especially significantly reducing the genes in group six and group four, respectively (Figure 3B).

In particular, we found that the abundance of GR, another chemosensory-related gene, in the saprophagous *H. assimilis*, *M. cinxia*, *V. tameamea*, and *V. atalanta* was significantly lower than in the nectarivorous *D. plexippus* (Table 1). GRs formed four conserved lineages in the phylogeny, and were annotated as potentially being sugar, bitter, CO2, and unknown function receptors. Despite a lower number of GRs identified, *H. assimilis* possessed more bitter receptors than sugar receptors, which was consistent with its saprophagous habit. Meanwhile, *D. plexippus* putative sugar GRs exhibited species-specific small expansions that could reflect its ecology as a flower nectar butterfly. A further analysis based on the branch-site model under PAML detected 1175 GR sites, one of which was under positive selection (in residue 996, *p* < 0.05) in *H. assimilis* (Figure 3B). The most frequent non-synonymous substitutions in this site were Phe to Ile, Phe to Ala, and Phe to Thr, which are generally conservative in most lineages, and can lead to the diversified selection of GRs in *H. assimilis*.

## 3. Discussion

Saprophagous habits have been observed in butterfly species, beetles, hornets, and flies [5]. However, little is currently known about the related genetic mechanisms. Our study provides unparalleled knowledge of butterflies’ genomic signatures behind saprophagy. We assembled a high-quality chromosome-level reference genome of the male *H. assimilis*, the representative of saprophagous butterflies in China. The final assembled genome was 412.82 Mb, and we anchored 98.11% of the scaffolds onto 30 chromosomes, consistent with the patterns of karyotype in *D. plexippus* [26,27]. Using a combination of synteny, Hi–C interaction, and sequencing coverage analysis to exclude an error in genome assembly, we documented a Z-autosome fusion event in a male *H. assimilis*. This discovery of Z-autosome fusion in *H. assimilis* creates novel opportunities to address the rates of molecular evolution, the evolution of dosage compensation, the pattern of allosome divergence, and many other essential questions in sex chromosome biology.

To clarify how saprophagous butterflies can avoid the adverse effects of diets, we found that gene families involved in alcohol metabolism, nitrogen, heterocycle, and the aromatic compound catabolic process significantly expanded in the saprophagous *H. assimilis*, *M. cinxia*, *V. tameamea,* and *V. atalanta* (Table S6). These were consistent with the characteristics of rotten food, that is, low sugar concentrations and the presence of fermentation products (ethanol and acetic acid) [5,9].

*H. assimilis* is particularly fond of rotting foods, which led to numerous opportunities to expose it to microbes and pathogens. Thus, we paid attention to the detoxification gene family. We annotated 80 P450 genes in *H. assimilis*, of which most CYP genes belong to the CYP4 clan, followed by the CYP3 clan. CYP4 played a crucial role in insect resistance to pathogen invasion and environmental stress, and CYP3 was generally involved in the detoxification and metabolism of exogenous substances [28,29,30]. The expansions of these two clans may benefit *H. assimilis* in exogenous substance resistance and survival in extreme conditions.

The chemosensory recognition system mediates feeding behaviors [22]. Niimura et al. found that the OR gene loss in primate evolution was possibly linked to anatomical changes in sensory systems and dietary transitions [31]. We identified the OR and OBP genes in the *H. assimilis* genome and compared them with those in other Nymphalidae species. We observed a reduction in the number of OR and OBP genes in *H. assimilis*, *M. cinxia*, *V. tameamea*, and *V. atalanta* compared to *D. plexippus*, because odor information is essential for nectar foraging, but less so for sap foraging. Notably, the number of candidate GRs identified in the *H. assimilis*, *M. cinxia*, *V. tameamea,* and *V. atalanta* genomes dramatically reduced compared to *D. plexippus*. GRs can be categorized into sugar, bitter, CO2, and unknown function receptors through differentiation in function [32]. Some studies found that sugar receptors can influence the host plant search and the oviposition behavior of female *Cydia Pomonella*. Moths usually use CO2 as cues to detect the floral food source, and CO2 receptors play an essential role in this process [33]. Bitter receptors mainly perceive the large variety of toxic substances that evoke aversive behaviors in caterpillars and moths [34]. *H. assimilis* bitter receptors were more numerous than sugar receptors, consistent with its saprophagous feeding behavior. Interestingly, we found a positive site of GR selected in *H. assimilis.* Whether the replacement to phenylalanine affects the combination of GRs and various taste substances in food needs further verification.

## 4. Materials and Methods

### 4.1. Sample Collection and Sequencing

Three male adult *H. assimilis* individuals (NCBI txid 378390) were collected from Hu county (34°09′00.00″ N, 108°32′00.00″ E), Shaanxi province, China, in June 2021. Two adult individuals were sequenced on an Illumina X-ten (Illumina HiSeq XTen, San Diego, CA, USA) and a Nanopore PromethION (Oxford Nanopore Technologies, Oxford, UK) platform [35]. All sequencing tasks were commissioned by the Novegene Technology Biological Company (Beijing Novegene Technology Co., Ltd., Beijing, China). For details of the specific operations and the use of kits, we referred to the company’s official website (https://www.novogene.cn/tech/service/, accessed on 28 August 2021). In brief, the short-read Illumina sequencing library was obtained using ~5 μg of DNA using the standard sequencing kit protocol (NEBNext Ultra DNA Library Prep Kit for Illumina, New England Biolabs, Ipswich, MA, USA). The nanopore long-read sequencing library was constructed using approximately 10 µg of DNA and the SQK-LSK109 Ligation Sequencing Kit. The retrieved library had a mean DNA fragment length of approximately 20 kb. The Hi-C DNA library preparation was constructed following a conventional protocol [36]. Before the Hi-C DNA library construction, formaldehyde initially processed the extracted cells by chemically fixing the cross-links of intracellular proteins with DNA and DNA to preserve the interaction relationships that maintained the structure inside the cells. Then, after treatment with MboI restriction endonuclease, the produced DNA fragments were used as inputs for the conventional DNA short-read library constructions, following the standard procedures. Using ~30 μg of DNA, the Hi-C DNA library was eventually constructed with the NEBNext Ultra II DNA Library Prep Kit (New England Biolabs, Ipswich, MA, USA) and then sequenced on the Illumina HiSeq X-ten platform.

### 4.2. Genome Assembly and Assessment

Before assembly, strict quality control was performed on the raw Illumina and Nanopore sequencing data using Trimmomatic v0.39 [37] and Nanofilt v2.3.0 [38], respectively. We removed the low-quality reads (Q30 < 90%) or those that contained more than 5% unknown bases. Environmental microbial contaminants were removed by deleting sequences with hits in the GenBank Env_nt database (ftp://ftp.ncbi.nlm.nih.gov/blast/db/, accessed on 14 September 2021). GenomeScope v2.0 and Smudgeplot [39] were performed to estimate ploidy and heterozygosity using Illumina short reads.

A de novo genome assembly was carried out using the Nextdenovo v2.5.0 software with the nanopore long read length cut-off and seed length cut-off values set to 12 Kb and 20 Kb, respectively [40]. The raw assembly was polished using Nexpolish v1.4.0 [41] with all Illumina reads for three rounds. Then, the haplotigs were removed using PurgeHaplotigs v1.1.3 [42] with default parameters. We mapped the Illumina reads to the above-polished assembly using Minimap2 v2.17 [43]. We used the ALLHiC pipeline [44] to conduct chromosome anchoring based on the Hi-C sequences with default parameters (-e AAGCTT -k 30). Finally, we applied RagTag v1.1.0 [45] to correct the contig orientation and move the suspicious fragments into unanchored groups by visually exploring Hi-C heatmaps. We conducted the synteny analysis with the reference genome of *M. cinxia* (GCA_905220565.1) and *D. plexippus* (GCA_009731565.1) using MCscanX [46]. The graphical view of the consensus map and other basic genome information, including the GC content and DNA variations, was displayed using CIRCOS v0.69–9 [47]. Whole genome completeness was assessed using BUSCO v5.2.3 (lepidoptera_odb10 database), which benchmarks universal single-copy orthologs [48].

### 4.3. Repetitive Elements, Gene Prediction, and Gene Functional Annotation

Repetitive elements were identified using RepeatMasker v4.0.7 [49]. Then RepeatModeler v1.0.11 [50] was used to construct a de novo repetitive element library, which was then used to predict repeats with RepeatMasker v4.0.7. Protein-coding genes were predicted with the masked genome assembly using a combination of ab initio, homology-based, and transcriptome-based prediction methods. The transcriptomic data were initially used in PASA v2.5.1 [51] to search for gene models, which were then applied in Augustus v3.3.1 [52] for the ab initio prediction. For the homology-based method, we downloaded gene sets for three related species in the same family: *V. tameamea* (GenBank accession: GCA_002938995), *Heliconius erato* (GenBank accession: GCA_018249695), and *D. plexippus* (GenBank accession: GCA_009731565). These homologous protein sequences were concatenated and imported into GeneWise v2.2.0 [53] to search for genes. Pseudogenes were filtered based on correct translation and the presence of mature stop codons. In addition, a concatenated ab initio and homology-based gene prediction pipeline identified genes using BRAKER v2.1.5 [54]. Finally, all gene prediction results were merged using the Evidence Modeler v1.1.1 [55]. The functional annotation of the gene set was conducted by querying the protein sequences against the InterProScan v5.41–78.0 [56] and NR protein databases (blastp v2.2.26 [57], with an E-value cutoff of 1 × 10^−5^).

### 4.4. Phylogenetic Reconstruction and Gene Family Analyses

The gene families were identified using the Orthofinder v2.3.8 [58]. The protein sequences of *H. assimilis*, along with the other 14 species, including *Aricia agestis*, *D. plexippus*, *H. erato*, *Leptidea sinapis*, *V. tameamea*, *M. cinxia*, *Maniola jurtina*, *V. atalanta, Pararge aegeria*, *Brenthis ino*, *Pieris rapae*, *Zerene cesonia, P. xuthus*, and *P. bianor*, were used to search for orthologous gene families. All single-copy orthologous genes shared across all genomes were selected and aligned in MAFFT v7.4 [59]. We used RaxML v8.2.12 [60] with default settings to build a maximum-likelihood (ML) phylogeny with the concatenated sequences. Based on the ML tree topology, the divergence times and nucleotide substitution rates were estimated using R8S v1.81 [61]. From the website www.time-tree.org, we selected three calibration points: the divergence between *B. ino* and *H. erato* (50.7Mya [62]), *Z. cesonia* and *P. rapae* (72.0 Mya [63]), and that between *P. aegeria* and *M. jurtina* (37.0 Mya [61]). According to the divergence times and phylogenetic relationships, café v4.2.1 [64] was used to determine the expansion and constriction of orthologous gene families. We used the significant expansion (*p* < 0.01) gene families for the GO enrichment analysis. The enrichment of GO terms was conducted using KOBAS v3.0 [65].

### 4.5. Positive Selection Analysis

Positively selected genes and sites were identified using the branch-site model in PAML v4.9e [66]. We used the M1A vs. M2A and the M7 vs. M8 models to explore sites that evolved under positive selection on specific branches. We used the likelihood ratio test to calculate the statistical significance of these models. Using the Bayes Empirical Bayes estimation [67], the branch-site model was also used to detect amino acid sites that were likely to evolve fast in the appointed branch.

### 4.6. The Evolution of P450, OBP, OR, and GR Gene Families Analysis

We performed a manual curation for each gene of the families that we addressed in this study. We identified detoxification genes in the genomes using TBLASTN v2.2.26 [68] searches with known *Bombyx mori* and *P. rapae* as queries. To eliminate potential pseudogenes, any sequence with predicted start and stop codons was discarded if it was shorter than its hypothetical product length by 25%. We selected only complete and nearly complete (>250 codons) P450 genes, where the conservative heme-binding motif Phe-X-X-Gly-X-Arg-X-Cys-X-Gly was present. For the OR, OBP, and GR genes, we identified this class of genes in the genomes using TBLASTN v2.2.26 [68] with known receptors of *H. melpomene* and *D. plexippus* as queries, followed by iteration. Gene structures within the genomic loci with significant hits (E < 10^−5^) were predicted using GeneWise v2.2.0 [53] with the pseudogene masked. We carefully checked the multiple alignments and removed poorly aligned regions before the phylogeny analysis. All phylogenetic trees were constructed in MEGA7 software [69] using the neighbor-joining method with 500-fold bootstrap resampling. Tree images were saved as PNG files and edited in ITOL (https://itol.embl.de/, accessed on 20 October 2022) [70]. All branches were manually collapsed to >50% bootstrap support.

## 5. Conclusions

In conclusion, we assembled a high-quality chromosomal-level genome for *H. assimilis*. We found that the expansion of the detoxification gene family and the contraction of chemosensory-related genes may be related to its saprophagous feeding characteristics. We raised the possible genetic specificity of *H. assimilis* in its saprophagous feeding characteristics. The high-quality assembly genome of *H. assimilis* also serves as a resource for further biological studies.

## Figures and Tables

**Table 1:** Comparison of detoxification and chemosensory gene families between the saprophagous butterfly *H. assimilis, M. cinxia, V. tameamea*, and *V. atalanta*, and the nectarivorous *D. plexippus*.

Family	Clan	*H. assimilis*	*M. cinxia*	*V. tameamea*	*V. atalanta*	*D. plexippus*
P450	Clan 3	23	36	28	30	33
	Clan 4	39	23	20	32	23
	Clan 2	11	9	8	7	13
	Mitochondrial	7	10	11	18	9
OR		33	43	43	52	57
OBP		20	25	25	29	32
GR		6	17	7	7	29

Note: P450: cytochrome P450; OR: odorant receptor; OBP: odorant binding protein; GR: gustatory receptor.

## Data Availability

All sequence data were deposited in GenBank under BioProject accession no. PRJNA793373.

## Supplementary Materials

The supporting information can be downloaded at: https://www.mdpi.com/article/10.3390/ijms24032087/s1.

## Abbreviations

OR: olfactory receptor; OBP: odorant-binding protein; GR: gustatory receptor; GO: gene ontology; CYP: cytochrome P450; Ka: non-synonymous substitution rate; Ks: synonymous substitution rate.
