# Supplementary material for: The Chromosome-Level Genome of Hestina assimilis (Lepidoptera: Nymphalidae) Reveals the Evolution of Saprophagy-Related Genes in Brush-Footed Butterflies
Source: Int J Mol Sci. 2023 Jan 20;24(3):2087. doi: 10.3390/ijms24032087 (PMC9917059; doi:10.3390/ijms24032087)
Supplement: Supplementary file 1 [file ijms-24-02087-s001.zip › Supplementary material.pdf]

Supplementary Materials

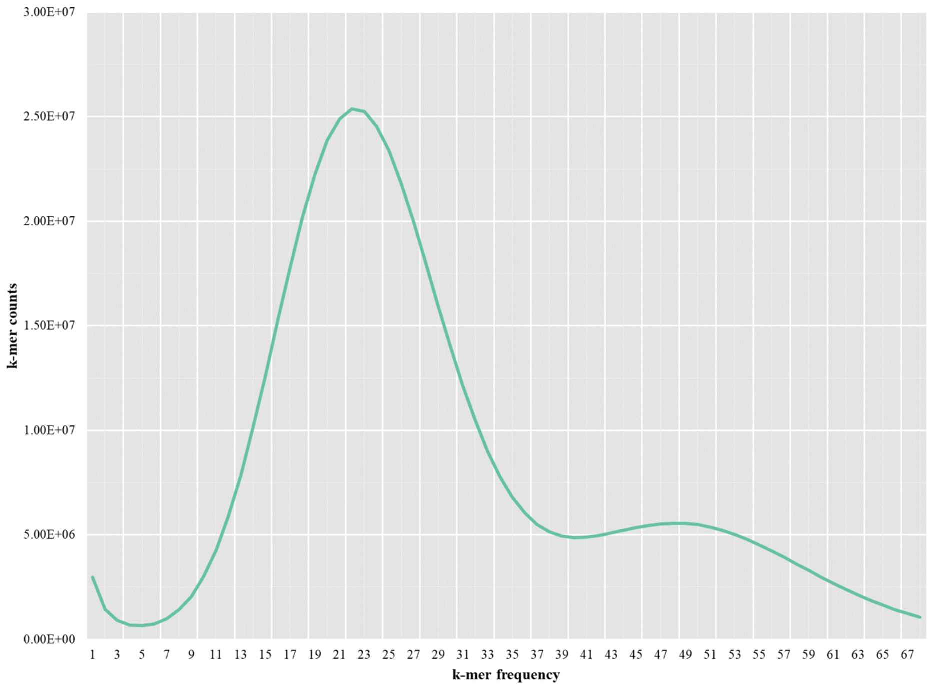

Figure S1 The genome size estimated based on the k-mer analysis.

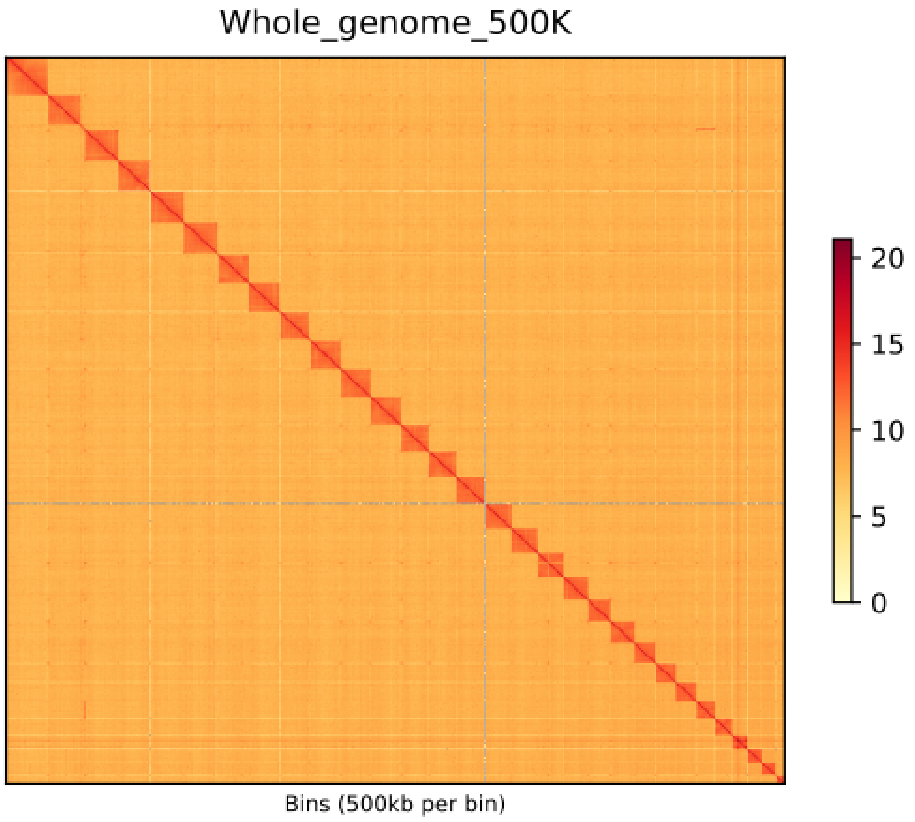

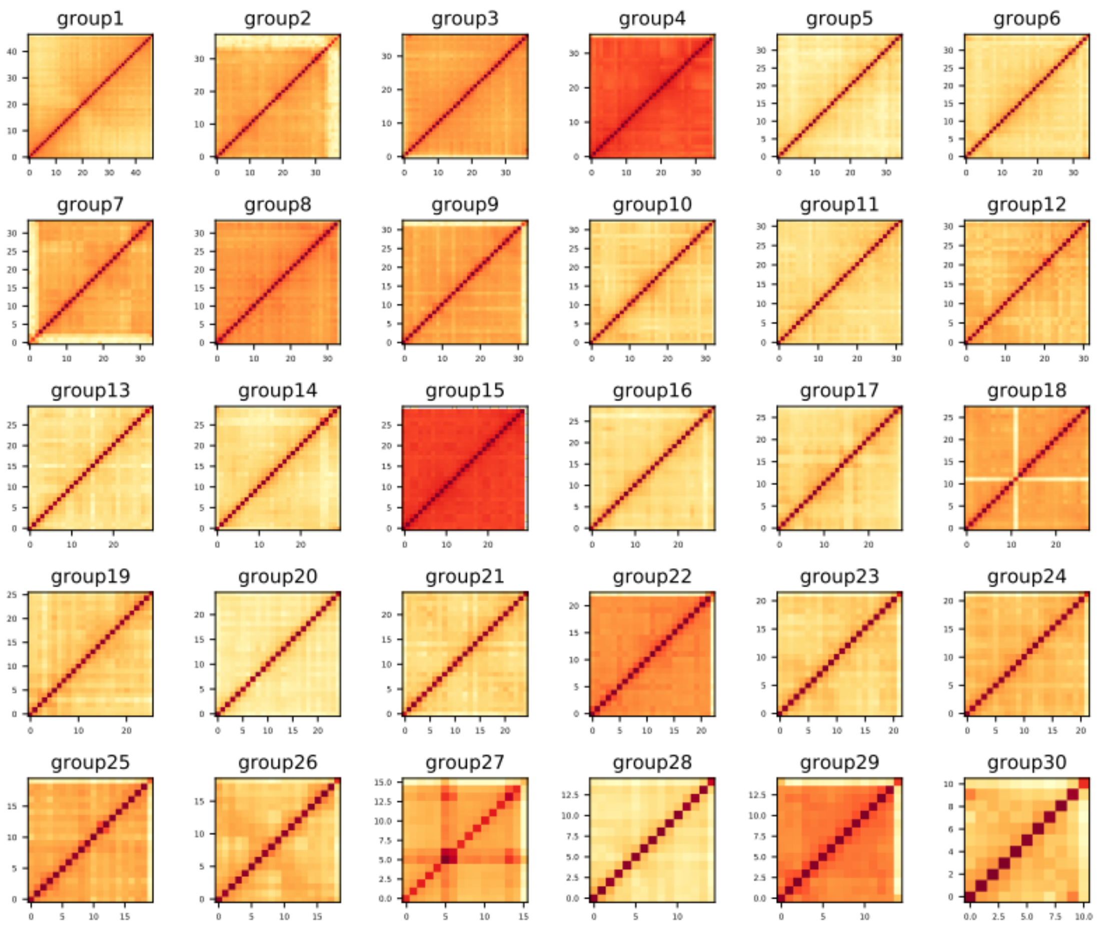

Figure S2 The Hi-C chromatin interaction map of *H. assimilis.*

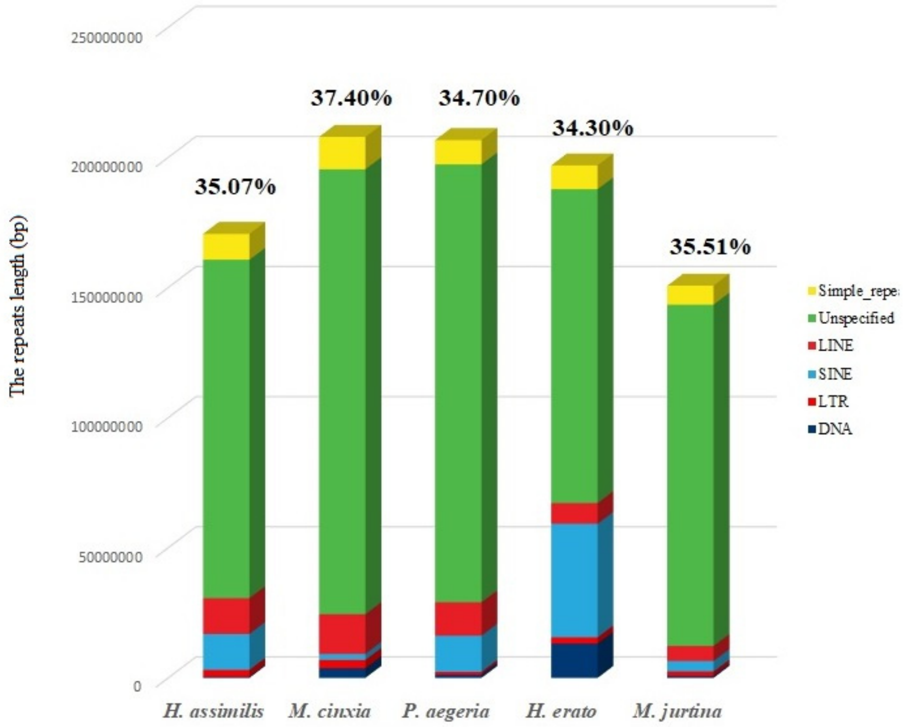

Figure S3 Proportion of each repeat class in the genomes surveyed. Numbers above

the bar plot correspond to total repeat length percentage in each species. The repeats length ranges from 35.07% in the genomes of *H. assimilis* to 37.4% in the genome of *Melitaea cinxia* are shown.

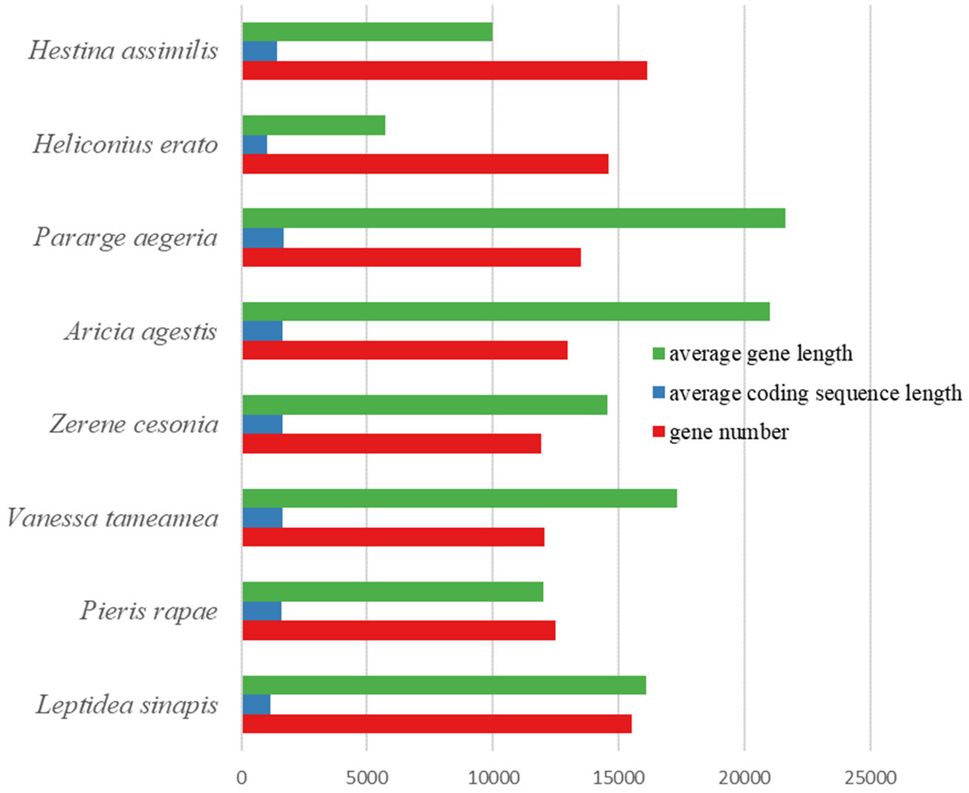

Figure S4 The comparison of gene number, average coding sequence length, and average gene length with other lepidoptera species.

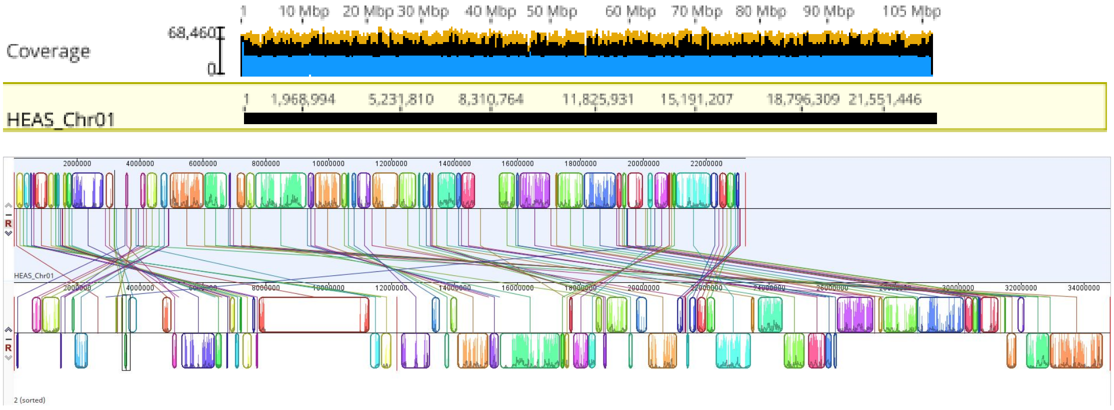

Figure S5 Normalized male coverage along the length of chimeric scaffolds for chromosome 1 of *H. assimilis*. Coverages are plotted as sliding windows (width = 64 kbp, step = 10 kbp) of median base pair values.

Table S8 Amino acid sequences of cytochrome P450 in *H. assimilis*.

Cytochrome P450

>HaCYP1
MLLIIVILFLISIYFYTTRNYSYWSKRNVKYETPLPIFGNHLKVFLGLKSLAMIS
NELYTKYSNEKVVGYFRGAAPELLIRDPDIVRDIMSADFAYFYPRGIGRNMKN
EPLLRNILHSDGDLWKLLRQRLTPVFTTSKLKSMFPLIIKCTEKLKNLGEEIVCE
GGECDAHDLMARFTTEFIGTCGFGIEMDVISKENSIFRILGKKIFERSLKDVLK
FGVWDVFPIFRSMIRIMDEKLYKTISGIVMKIFEQRNFKPSGRNDFIDLLLDLA
GKGKIVGDSIEHVNPDGSPKEVEVKMDIDLLIPQVFMFFAAGFETSASITSYTL
HELAFSADIQRKVQIEIDQVLSRYDDKLCYDALAEMTYFQMVLKESMRLHPA
ACVLNRVCAKTYQISQLGITIDPGVRVIIPVQALQNDKLYFESPDEFNPDRFIDE
IDSRHKYVLLPYGEGPRACIGARMGQMMSLAGLAALLRKFSVEPSPKSRRILQ
MNPRQNVVQAALNGIPLKLKLLKKKAAARTASRNLTGGGAIEDLPELSHVEK
RIITLMGGEGFATGDRHLQIQALEPIQEQESPSILQPMLESSSTCSIQIYLYLTNPS
ITTFSETLKFDGNNGEVSKTIYFYFTRNQNYWSVRNIKCDRPLPLFGNHLLNV
LGIKSIATITTELYNKYPNEKVVGYYIGTQPQLIVRDPEIARDILNVDFAHFHLR
GLGRDHNKEPLLKNIFNADGDSWKLLRQRLTPAFTTAKLKGMFPLIVKCAEK
LHGLGDEIVAKGGECDVRDLMARFSTEFIGACGLGIEMDTINNENSVFRKIGK
EIFLRSLKDVFLLGVWDIFPEFRPMLHLFDKNLEKVLIEIITKVFEQRNFKPSGR
NDFVDLLLDLAAKNKIKGDSIEKRNADGSPKEVELEMDTLCLIAQVFVFFAAG
FETSSSSTSYTLHELAFEPDIQRDVQEEIDKVFAKYNNKLCYDAVAEMPLLERC
FKESMRKFPPLGILNRVCANKYTISKIGVSIDPGVKIIIPVQAIQNDDKYYEKPD
QFKPERFGADDVTTTQKYIYLPFGEGPRACIGARLGLMQSLAGLAVILQKFSV
EPSEKTTRKLKINPRLNIVQGVMDGIPLKLNLRNK
>HaCYP2
MTLFVVRSYKNAYRKPVSNFPPGPPSLPIYGAYWILLFREFDNLAVSLYKLAK
DYNTKILGIYLGIFPTIVINDSKLIKEGLNCENFDGRPDIIVGRLRSFWKRLGIFF
TDGYFWHVQRRFSLRYMRDYGFGRRDESLETVIANETKEMIDMALNGPKYP
AEKELVKGDLIHMQHFFAVPFINGILHIFTRSTLPRSDYHILWEMARYTLMFQR
GSNDLGGALSLTPWLKDLLPNYSGYTNLRKGNQKLLDFFGKLIKETIKTQEDS
HDRHFLDCYIRKTKEEQKSTGRTTFAEDQLQLVCIDYMFPSATGTESMLTILIE
RILLQPEVQDKIHEEIDRVVGRDRLPTLDDRQNMPYTEACLREIMRFETLVPLG
VPHRTMKDTKFGGYDIPENTLVSFNYYSLHNDKEIWGDPENFRPERLIENGKL
QLSKDKSLPFGAGKRLCAGETYARQAMFQVFAGFMQAFHVSTADGPPSLPIY
GAYWVVLIREINNLAGSLYKLARDYNTKVLGMYLGTYPTIVIDDPKLIKEGLN
CENFDGRLDIILGRLRSFWKRLGIFFTDGYFWHVQRRFSLRYMRDYGFGRRD
ESLETVIANETKEMIDMALNGPKYPAEKELVKGDLIYLQNFFAVPFINGILHIFT
RSTLPRSEYHVLWDLARNTLMFQRGSNDLGGALTITPWLKDVLPNFSGYTNL
CKGSQYLLDFFGKLINETTNSQEDSHDRHFLDCYIRKMKEEQKNGGRTTFTED
QLRLVCIDYMFPSATGTESMLTILIERILLQPEVQDKIHEEIDRVVGRDRLPTLD
DRQNMPYTEACLREIMRFETLVPLGVPHRAIRDTKFGGYDIPENTVVAFNYYS
LHHDKEIWGDPENFRPERFIENGKIQLSKDKSLPFGAGKRLCAGETFARQAMF
QVFAGFMQAFHVSTADGEPMKKPARRIQGIITTLPEFWIRVTPRT
>HaCYP3
MRHSVCLRWPLRRFERSFGTTRHAAQASDGSDIGNLSSRHTCPAHSRARSSHA
VASVLETLSPTVKSWEEVPGPKPLPLLGNTWRFIPYIGGYSVEHVDQVCLSLK

KQYGKCVKMAGLLGRPDMLFVFDASEVERVFRGEDAAPHRPSMPSLNYYKH
TLRKDFFGAEKNCAGVIAVHGDSWAAFRTKVSRVALSTGAAAQYTEQVGEVA
DAFVTRIRDIRNSKLETPDDFLNEVHKWSLESLGLIALDTRLGCFESCEGSESQ
RLIDAVHTFFLCVGELELRAPWWRIYPTTMFKRYVAALDTILSVTLSHVERAL
QECQVNGNKSLLQDLVTAAGSRVAAVAALDMFLVGIDTTSNAVASTLYQLSLN
PRVQEKLYKEITGVLQGRPLKAGDISQMPYLKACIKEVLRMYPVVIGNGRQLT
KDTIICGYNIPKGLLDGPDLLQSLPGIMYRFREGAVAVTADIKEMFLQIKVRPA
DQPAQMFLWRGRDRENPPKRYKMTSLIFGASSSPFIAHSVRNKNAQDFIHTHP
AGYEAITRNHYMDDFVGSFADKEEARRTVDEVNYVQQQASFTLRGWDTNER
GVLTNIPPELHSSLPTHLMMGLDASRTLGLIWDSRRDELGFNTNMSRVHEEVK
SLSRAPTKRETLSAVMSIYDPLGFLSPYTIVAKIILQSLWKTDVGWDDEIPQELA
ERFHEWMQGLETIKTLRILRWYGVSKNDVRRELHIFCDASELAYAAVAYWRIE
KSDGTVAIALAAGKAKTQVIFQHYVMGNSEEYFSNASEFRPERWINRSTYKQ
HPFASLPFGFGKRMCLGRRFAELEMHVIICKMVQAFQMEYHHEPLEYHVHPM
YTPNGPIRIKLLER

>HaCYP4

MKVGPKKIAFLINPEDVEILLICTKSNRKAYYYDFLRPWLSEGLLLSYGKEWH
QRRKTLTPAFHLNILRHFNSVLIERSNSLVKQLKTEINNSKTDMFSYLTDFSLNS
ICETAMGTVLDEKESEIGKNYKNAIHKLCTYIYYRAHKIWLYPEFIFNLTRVGR
DQKRLLQLIASFRNEVIEKRRKSNNYKTISTELMNEDLDDMFVYKKNRFAML
DLLLEAESEGKIDPEGINEEVDTFIFAGYDTTATALQFVFLLLANHNDAQGYDT
TATALQFAFLHLANHKDAQDKILEECNRILSSNDRKPTMNDFAQMKYLEACIK
ETIRLYPPVHIMSRTCEQPLQFKNFKCPAGTEISIPVFMLHRRSDQFVDPLEFRP
ERFLVEPTWHPFSYIPFSAGQRNCIGQKFAMLEMKLAISAVLAEYRLVPVTKPE
DVLMSMDMGYDTTATALQFAFLHLANHKDAQDKIVEECNRILSSNDRKPTM
NDLSQMKYLEACIKETVRLYPPVHIMSRTCDQPLQFKNFKCSAETEVVIPVFA
LHRRSDQFVDPLEFRPERFLVEPTWHPFSYIPFSAGQRNCIGQKFAMLEMKLAI
SAVLAEYRLVPVTKPEDVLMSMDMVLRTKDPIYVKFEKRNNKTT

>HaCYP5

MIIVIIISIILCFYWIYWVAGTWRMDKATASLPTPPSVPIIGNAMLFIGNTEKILK
NLEDIAALAFEHKGVVKLWLGPKLYIAIGNPQDAQVVLDNCLDKDVVYRFLQ
PWLGQGLFIAPLALWKIHRKVLLPVFHNKIIEEYLANISKQANVLLERLFEQSG
KPKFDILPYITACTLDIVFETAMGERMDVQHSPDTPYLRARHTVMTIMNKRLF
KVWLQPDCVFNVTPYAKQQYESIDFTHKFTDEVVRKKRTEFETRSTSKTDED
KDRKPRAVLDMLFDREIKFTNTQLREHIDSITIAGNDTTALVIAYTLVLLGIHQD
VQEKVLQEQIMIFGHVKKGATKEDLQKMNYLERVIKESMRLYTVVPIIARNID
KEIHLPHSGVTIPAGVGAVVGAFAIHRSKEVWVPNANEFDPDRFLPENSVDRH
PASFLPFSLGSRNCIGREQHWEEGDPVWVRDFRKGRKWMKGVILLRKGFNSY
EVRTDDGLLWLRHTDQIRSCATRDSTPEGAANASERKPCVAPQAALTGRNFG
MIIMKSIISSVIRSYKIEADDIGPLKIEMLLFPINGHQVKVTKR

>HaCYP6

MLLNNLRYLQPTTRKFVVKNGNGHIWYNIRRQTTKPLVINDDVTKSAEKYDK
IDPISDITPVPRIVPMVLNNREPVVLPFNDIPGPKSLKYFSMFRNSITEIGTQLTA
GFLTFGTFLANRRPIPNFSSLFDKYGPVVRFVSPVGSDIVLINHPDHIQKVFSME

GEYPVRSTLESLERYRNEHKNHIFGGLYTGNGQDWVRQRSVVHSPVNNAVFQ
HAQNVYHTCEKFTQKVYNIRNYQDEISKDLYKELHKWAFDCMGLILFSKDFK
MLETELIYNQCDSSWMYNSLDKATDAIIKCETGVHLWKFFTTPAWYSLVKHC
DILDSLIGKHVLDIEQEISTNTQDDNATKAGSLTSAILLSEEKFKAEDVATILMD
MMLIGVNTITSSMAFLLYNIAKHQKCQRLLYKEVGNLYPDMIVKDVENFKGN
TPYLQACIKETFRLVPPIPLITRILSRNITLDNYNIPRGTLIIMSTQDASLKEGNY
DDAKKFRPERWLKPEAQEYHAFASIPFGFGARKCVGQNISETMLSLLTIKVLQ
KYRLEYHYGDVQPSRGFITRPNRPLKIRFIDRM
>HaCYP7
MAEELAAVVGKGNVSLEKYINSYSLDTSCDTTMGDSVKSQRQLEHPFLVAFT
KYTENLMDRMCKPWLHIGAVYKRQAVYSKQVEYKKTIVDFVEKNIAKTRLL
NEENKHDEGESCDSMEEEGSKRIKTFLELLTEHSGYNDLELREETLILILAATE
TTALTSGFTGVLLARHPDIQEKVYEEIREVFENSSRPLSIDDLNNLKYLDAVIKE
TMRLYPPGPALMRTCDSTVTLPSGLVLPKGSNVIVNIWAIHRNPRYWGADADE
FKPERFLNASREQLAAYMPFSSGPRSCLVISEPEAANFILKSCLDKGRLTSFARH
LFGNGSIFASASTINANIQSFLESLTEHSGYNDVELREEALILLLAATETTALSSG
YTCVLLAHHADVQDRVYQEIYEVFGDSSRHICSDDLNNLKYLDAVVRESLRL
YPPAPILARGCHSDVRLPSGIVLPKGTNTIINTWAIHRNPHYWGEDADEFKPER
FINVQREQLAAFLPFSSGPRNCLGYHYAMLTMKTNLATLLRRYRIVPATSFKY
DDQNPLRVKFSVTMKHVHEYEVQLEHRLRH
>HaCYP8
MTVVEDIHPYTLNTTRLMFYPLVLLATTLWLLYRWQQQSRLYKLGNKIPGPSA
VPLFGNALLALGKRPEQLLSLALQYADKYGTVVRGWLGTKLIIFLSDPDDVE
VILNSHVHIDKASEYRFFKPWLGEGLLISSGEKWRSHRKMIAPTFHINILKSFV
GLFNQNSKNVVDKMKGEVGKEFDVHDYMSGVTVDILLETAMGITKDTQGN
DGFDYAMAVMKMCEILHHRHYKFWLRFDFVFKFTAFFEKQKKLLGIIHGLTN
KVIKNKKEKYMLNKSKGIIPPTLEEEIRASSSENETGLANVKTLADTVFKGYR
DDLDFNDENDVGVKKRLAFLDLMIESAQNGTNKITDHEIKEEVDTIMFEGHD
TTAAGSSFVLCLLGVHQDIQTKVYDELYSIFGDSDRPCTFEDTLSMKYLERVIL
ETLRMYPPVPIIARKLKRDVKIATNNYVLPAGSTVVIGQFQVHRNPKYYSNPN
VFNPDNFLPENTQNRHYYSYIPFSAGPRSCVGRKYAILKLKILLSTILRNYKMV
TNITEDQYVLQADIILKRNDGFRVRIEPRKRVPSTV
>HaCYP9
MGGGDRLTPVLHLLYNIYGVWLRNLIIMILFIWCAVLVSVLLLYCRKTYSRFSK
HGVKNEKVVPLFGNMLNAVLRQEHFNEGFNRLYNDFPEERFVGRFEFTKPSIV
LRDLDLIKKITVKDFEYFLDHRGLVDETNEPLFARNLFSLKGQEWKDMRSTLS
PAFTSSKIKLMVPFMEEVGDQMIATLKAKIKDSDTGSIELDCKDLTARYANDVI
ATCAFGLKVNSHTDVDNHFYKMGTTVSNFKFRQLILFFAMAACPSVIKVLKL
KLFEKNFNDFFIDLVLNTMKDRKTRNIIRPDMIHLLMEARKGQLTHDKKSVNG
HQDTEFAAVEESSIGKKHTDRVWSDVDLIAQAVLFFIAGFETISTAMSFALHEIA
LHPEIQERLVKEIKDHHAKNGEKMDFNSIQNMTYLDMVTSEVLRLWSPALAL
ERTCKKDYNMGKPNSKATEDFIIKKGETVWIPTWGIHRDPKYFPNPEKFDPER
FSDENKHQINTMAYMPFGLGPRNCIGSRFALCELKVLLYQVLLHLEVSPSKKT
CLTGKLSTKSFNPRLEGGHWLNFKIRE

>HaCYP10
MVIRLLVMIITKKKKAISFYEQFSVTLSGLLFPPVRQEENRSDGRERENNGTEL
SVFFLECFRMFLLTIVSVVLCFVLWIYIRWLRYKRYWADRGVPHLPPVPILGSL
TFLQRENMNKWFRGMKEQFDSPYVGIWVFWRPGLIVNSPEIARNILVKDFDN
FRNRLLGSGESDPIGSLNIFTSNDHVWTSMRRQLTGIFTAAKLRNLQRFTRSKA
EELIQRIKNDQNIKIDLKCICTDYTTDVIGTAAFGVRSNATLTGGGPLRDVTNSF
SKYSIYRGISWCSIFFFPELVDIFRFTFFPSSSTKYFKKIYHTVTLQREVNQNDTE
PRDLVDTLLKIKRDNKSYSEEMIIAQAAILLLGGYETSATLMTFIIYELAFNHDI
QEKLYQELVEARDKNGSDEFDIQVLTDLTYLDCVIKEGLRKYTTMGWLDRVA
TNDYKIDDKLTIEAGTVVYINSIGMHYDPKYFPEPDKFIPERFLPENKNNIQPFT
YMPFGEGPRFCIGKRFGLMTLRFALTLLLLNFNIRPFPDSPKPSDIKFNTIGIFLA
PGETLYVDFVPRKE
>HaCYP11
MGYAATESVAVSNTWAATNLFYVLLVPAILLWYAYWRMSRRHLYELAEKIAG
PKGLPLIGNALEFTGGSPDIFRRVVERGNEYNKENAVKIWIGPRLLVFLFDPRD
VEVILGSHVHIDKAEDYRFFKPWLGDGLLISTGQKWRSHRKLIAPTFHLNVLK
SFIDLFNANSRAVVNKLKKESGVFDCHDYMSECTVEILLETAMGVSKSTQDQS
GFEYAMAVMKMCDILHLRHTKIWLRPDLLFNFTQYAKIQNKLLDVIHGLTKK
VIIRKKEEFKSGKKPSIVETETSDKESLSSKVTSVEGLSFGQSSGLKDDLDVDD
DVGQKKRLAFLDLLLESAQGGVNISDEEIKEQVDTIMFEGHDTTAAGSSFFLS
LMGIHQDIQNKVVEELDQIFGDSDRPATFQDTLEMKYLERCLMETLRMFPPVP
IIARHLKQDVTMPSNGKKIPAGTTVVIATYKLHRREDVYPNPEKFDPDNFLPER
SANRHYYAFVPFSAGPRSCVGRKYAMLKLKIILSTILRNFRVHSDLKESDFQLQ
ADIILKRAEGFKVRLEPRKRATKA
>HaCYP12
MKAHITWISSQAARVKSIKPKINMIILLIWVFVLVAALLLYFRQIYSRFSKYGVK
HFKVVPVFGNMLGIVFRRETMSDEMDRSYNTFSDERFYGRYEFITPMLVIRDL
ELIKKITIKDFEHFLDHRGLTDETVEPLFARNLFSLKGQEWKDMRSTLSPAFTS
SKIKLMVPFMEEIGEQMIRALKKKIKESENGSMNVDCKDLTTRYANDVIASCA
FGLKVDSFTEEENKFYEMGKTASTFKFKQLLAFFAVSACPAIAKRLKLKLFSN
QTRDFFIDLVLTTMKDREARNILRPDMIHLLMEAKKGQLSHDDKNETHQDTG
FATVEESSLGKKSIDRVWSDNDLIAQAVLFFVAGFETISTAMSFALHELALHPD
VQDRLVEEIKENHIKNGGKLNFSSIQSMTYLDMVTSEVLRLWPPGIALDRLCI
KDYNIGKPNSKSTEDYIVSKKGEIISIPAWSFHHDPKFFPDPTKFDPERFSDENK
HKINPTAYMPFGLGPRNCIGSRFALCELKVLLYQIILYMEISPSEKTRLPAKLSTE
SFNPRLEGGHWLKFKVRD
>HaCYP13
MRTKALVCSGARQQVQGATPDQYEPAPRRAAARRRPSPFDPGRPQSTQTTIYT
SRSNIMRLLIFVLRKLERERWGITMVNTRDLLAIDGAGGAAKVAKLFGHPDLV
FPFCAEESAKIYRREDSMPHRAAAPCLKHYKQELRKDFFGDEPGLIGVHGMP
WSKFRSKVSKALVAPEAAKAMVPALDDVAIDFVNRMEQILNHNRELPMDFLT
ELYKWALESVGAWALGTRLKCLSDEDTEAREIIKSIHGFFHSVPELELSAPLWR
LYSTVAYKTYVEALDSFRILCLKRLTDKGVCAKIAQSSGEKVATILGLDLLLVG
VDTTAAAAASTIYLLAKNSRAQRRLQEELDNRLPVGKTLNSKDLDQLSYLRA

CIKEALRIKPVILGNGRCIQSDAIISGYEVPKGSHIVFPHYIMSNEERYFPNPHEY
VPERWLRDKEHNTNGSSPTTDNNSNKTICEHAKAMEISEKQRMMGIHPFASLP
FGFGRRMCIGKRFAEVELQLLLAKVFHRYNVSWRYGDLTYSVTPTYVPNEPL
RFKMDVRKSEN
>HaCYP14
MITMLSNSKLLWGLWQVVSYCSSRTTMPLLLIVGMTFLAMRLINLVREIRKLP
PGPWSLPVVGYLPFLGVRHKTFLELARNYGALFSARLGNQLTVVLSDYKLIRE
AFRREEFTGRPTTPLMNILDGLGIINSEGRLWKSQRRFLHEKLREFGMTYMGN
GKKIMEARIKNEVHELIANLQCTEGAPIDANPLLALGVSNVICGITMSVRFSHG
DVRFARLNHLIEEGMRLFGEIHYGEYIPLYNYLPGKALIQEKVAKNREEMFAF
YQTLIDEHRNTLDINNARDLIDVYLIEIEKAKIEGKEGELFEGRDNELQLKQIL
GDLFSAGMETIKSSLLWMIVFMLRNPDVKRRVQEELDSVIGRERLPTIEDMPN
LPYTETTILETLRMSSIVPLATTHSPTKDVHLNGYRIPAGSQVVPLINCVHMDP
NLWQEPNKFNPSRFIDESGKIKRPEFFMPFGVGRRMCLGDVLARMEMFMFFS
SMMHQFDVQMEADVAPPSLEGTVGATIAPQNFRVKFITRAPPAPPAVILHDHP
HLRHVGAH
>HaCYP15
MSALLIIALCVFIIYKYVTKRTVTYRRANKYGENKIQELQPAPGPIPLPVIGSMH
LLGRNESPFQTFTELAGVYGDVYSLKLGTSQCLVVNSLDRIREVLNQNGKFFG
GRPDFLRFHKLFAGDRNNSLALCDWSNLQLRRRNLARRHCGPKQHTDNFSRI
GTVATFESVELIQNLKNITSSTTNSINIKPILMSTAMNMFTNYMCNVRFDADTD
VEFKKIVDHFDEIFWEINQGYAVDFLPWLAPFYKKHMEKLFNWSQDIRSFILS
RIVEQRETNMDMDGPEKDFLDGLLRVLHDDPTVDRNTIIFMLEDFLGGHSSV
GNLVMLCLAAAARDPEVAKNIKSEIDNLTQRKRAVTLADRSSLPYTEATVLEC
LRYASSPIVPHVATENANIAGYGVEKGTVVFINNYELNTSSKYWDEPEKFDPSR
FLEKTKVKTRRNSLCDSGMESDSEKSGQGLNQDEEVEIEVVSVKKNIPHFLPF
SIGKRTCIGQTLVTTMSFVMFANIMQEFDVAAVDKNDLRQKPACVALPKDTYH
LYLLPRK
>HaCYP16
MDFFFVWLVTFVLGYWIFKKINEWKNLPPGPWGLPIVGYLPFLDRQQPHLTLT
KLSQQYGPIYGLGMGSIYAVVLSDHRLIREAFAKDVFSGRAPLYLTHGIMKGN
GIICAEGALWKDQRKLITSWLKSFGMSKHGVARDKLEKRIASGVHELIENIKE
TSGSSLDLSNMITHSFGNVINDIIFGFKYPSHDKTWHWFRQIQEEGCHEMGVA
GVVNFLPFVRFIYPSVQKTMEVLVRGQSQTHRLYASIIARRRKMLGLTVPKDA
VYAEHADLFNEHPEGFIKCVTYSKHVSITESHYFDPSVLIASEDECILDNFLNE
QKKRYENGEEGAKYMTDEQLHYLLADMFGAGLDTTSTTLSWYLLYIALYQD
EQEQVRQEILSVYPEDCQPDCSKLPYLMATVCETQRIRSIVPVGIPHGCLQDTY
LGNYRIPQGTMVVPLQWAIHMDSNIWEDPNEFKPNRWIDECGNLLKPQEFIPF
QTGKRMCPGDELSRMVSVGVITRLLRSFRIRLAGKPPTAEEMQGKVGVTLSA
PETLFICETL
>HaCYP17
MHVQQDPDDRITFENVPYPVQTLTQHRLRRRGRGPLFRTSRRAHTVGSAMLL
AYLILSCVLFGVLLFFQQRRNRNEPPLLSRALPIIGHAHLLAGSTADLWNFLKE
LILECVKRGGLTTFRLGPITLYALTDPDDFLTVANACLQKDNIYDFAKPWLGEG

LITGTLPVWKVHRKLLNPAFNQIVLDGFLDVFNKQARRLVNEFEIEVDKKSFD
HHTYIRNKSLETICLTALGVDLNEKSELNTQYINAVDQLFKIFMERTQKFWLHS
DLIFNWSVHKGRQDEFVKNFHNMSHTVLQKTRENYKNNKEEKGPKFKAFM
QLLLELTIEKDLLKDNEIREEVDTMIAAGYETSATSLTFCMLMIGSYPEVQEKV
VKELKEVLGDDDRDLTKQDFSNLIYLDAVIKETLRFYPVAPVVARYLDKDIKL
RDYTLYKGARCFMFIYGVHHSSIWGSDAEEFKPQRWLDSASLPKSPAAFVGFS
AGRRNCIGKAYALMFLKTSLAHVLRKYKLQGDHTKLELDLDIVLRPVSGHHI
SIQRRK
>HaCYP18
MIILVIWGTVLLAVLVPYLYQTYSKFSNQGVKNLKVFPFFGNKFKMFFRIETLA
DELDRNYETFPEERFVGRYDFTKPVVLVRDIDLVKKITVKHFEHFLDHRNFVD
EKMEPFFARNLLMLKGQEWKDMRSTLSPAFTSSKIKLMVPFMEEVGQQMTQ
ALKKRIRESKTGHIDVDCKDLTTRYANDVIASCAFGLKVDSFTDEKNKFYEM
GKTVTNFNFKQILYLFTILASPALARNLKLTIFSKDTKQFYMNLVLNTMKDRET
RNIIRPDMIHLLMEAKKGQLSHEDKEDNENYQDTGFSTVEESSIGKKTNAKV
WSDNDLTAQAVLFLIAGFETVSTATSFALHELAINPEIQERLVHEIKEHDVKNG
GKLKFSTIQSMTYLDMVISEILRLWPPGLGLDRMCVKDYNMGKPNNKSTKDY
IIRKGELISIPVWCFHRDPNYFPDPMKFDPERFSEQNKHTINPAAYLPFGSGPRN
CIGSRFALCELKVLLYQILLHFEVSPSEKTHLPAKLSTESFNPRLKGGHWLKFNI
RE
>HaCYP19
MSKAVLARQCLLSRPSRRQISTSSVRRTSTSPQRRNVAAAAIYNNYKKFSDIPG
PMALPIMKHHAHVMMIQRVTKALFPGSFHHTVGLGLLEGLRERYGDLVRLA
KGSRSRPVLYVFDPELMREVYESNMTEPPQWMRSPLEEQRKNAGTQCPMQR
DETEAVWAGIRTLLKDGAILRNYDKAFDDIAADMTRRLGELRHAENALNEEF
ETEVYRWAIETIGVMLFGIRLGCLDGAVHIPSEENRRPEKTSMDDHIKDLCSLS
TRCLTELNPAEQFVRCSLEIANESYLVRSEHTLRPDLTDDFLLKALHELNNDEL
RPEQTLLDKLRPLDRRILPLAADMFLAGVEPLAQTAVSMFYQLSLHAARQQR
AHDEVVWAKASRDEGLDIQELPYIASCAKEAMRLYPATGGVVRRSREELVVG
GYEVPAGVDIVLAHGVTSKLEEQWGRAKSFIPERWCSQAWEPLKASRAHSVA
SMPFGQTCPATGIVNKMLSTLATKIVEKYRLEWHGPNPNLVTTGVNKIQPPYY
FVLQNAS
>HaCYP20
MGRSLNSLKPYTRPFTIKNSRQATTGCPFSKRQRSQIAPAASLNEEIFGNAKPY
SAVPGPKPIPILGNTWRMVPVIGQFDISEFAKVTKSFLDKYGRIVRLGGLIGRPD
LLFVVYDADEIERMYRREGPTPFRPAMPCLVKYKSEVRKDFFGDLPGVVGVHG
EQWRRFRSKVQRPILQPQTVKKYVTPIELVTEDFIRYMENARDENGDLPHEFD
NDIHRWSLECIGRVALDVRLGCLSPNVTSDSEPQRIIDAAKYALRNVAVLELKA
PYWRYFPTPLWTKYVNNMNFFVELCSRYINEALERLKTKKVTSENDLSLLER
VLQSEGDPKIATIMALDLILVGIDTISMAVCSILYQAATRLKQQDKMAEEIRRVL
PDPSKPLTYADLDKLHYTKAFVREVFRMYSTVIGNGRTLQEDDVICGYHIPKG
VQVVFPTIVTGNMEQFVSNPEEFRPERWLENDGRLHSFASLPYGFGARICLGR
RFADLEIQVLLAKLLRRYRLEYHHEPLEYAVTFMYAPDGPLRLRMVERSS
>HaCYP21

MIYIIWVFILIAVLAVYFRQTYSHFSKYGVKNMAAVPFFGNMLRIVLQINHFGD
EMDRMYKKFKGERFVGRYEFVRPILVVQDLELVKKITIKDFEHFLDHRTFTDE
KNEPMFARNLFSLKGQEWKDMRSTLSPAFTSSKIKAMVPFMEEVGEQMIRAL
KKKLKESDSGGIDVDVKDLTTRYANDVIASCAFGLKVDSHTDENNQFYEMGR
IASTFNFRQMLILFLISAFPSITHILKLKVFSDSTKSFFVDLVTGTMKDRRTHHII
RPDMIHLLMEAKKGKLSHDEKSDTQDAGFATVEESSVGKKTVDRVWSDMDL
IAQAVLFFIAGFDTVSTAMSFVLHELAIHPEVQERLAQEIKEQDVKNGGKLDL
NSIQNMTYLDMVTSEALRFWPPAMALDRICVKDYNLGRPNSKATKDYIIRKG
DVISIPVWSFHRDPEFFANPNKFDPERFSEENKHNINPMAYMPFGIGPRNCIGS
RFALCELKVLLYQILLHIEVSPSKKTLLPAKLSPDTFNPRLYGGHWLKFKSRS
>HaCYP22
MIVEIIIFVITSIFVYFLYLYKWVHKFFDDRGIKYVPGVPIFGNVVKSTFLKNHV
VEDIDRVYKAFPGERYVGYIEGPSPIFLVRDPELIKTITVKDFDHFVDHKQFFSE
EIEPLFGGSLIMMRGEKWHDMRTTLSPAFTGSKMRKIMPFMTEISSNVIEYLR
DHVNEDIDIDDLMRRYTNDVIASTAFGLQVNSVKDKNNEFYMLGQNLFKFNF
FQRMYFFITTLCPNLCKKLGIQIFPAKTTQFFRNIVTSTMEYREKNKIERPDMIQ
LLMEAFKGSLKADNEGNDNNILAEDTFKPKAVQRQWTQNELAGQVFIFFVAG
FESSATSLVMAVHELALNPHVQDKLYQEIIKFKEEHGDVTYDNINSLKYLDCVI
NETSRKWAAALIMDRVCNKAYELPPPRKGAKPVQLKPGDVIYNVVNSIHMDP
EHHPEPEKFDPERFSDENKHKIKPFTFMPFGMGPRICIGSRFALLEIKILLYNLVL
NYKVVKCSKTTDPIELKPHAFTIQPKGGCWVRLEPRI
>HaCYP23
MVIYPIITLIFGVFIYIYYFLTRYNDYWKKRNVAYLKPSLLFGNYKEYILFRKCR
QKIAHEICQQFPNEPYVGTFYGTDPALIIKDPNLVKLVMAKDFYYFNHREVSQ
YTHKELLTQNMFFNGGDTWKIFRQNLTSLFSSAKIKNMFYLIESCAGCLENVI
NKEKTEDNIIEIKAVLARYTMDCIGNCAFGVNTGTLETNSPSNVFTVMGIKLFD
VSNYGGFRLYARSMWPNIFYTLGFTMFESDIHSFFTKLMTEVFESRQYDESSR
NDFVDLLMSWKKKNCITGDTIMSLKTGDKTTISLNVDNKLLISQCVLFFAAGF
ETTATTTSFLLYELSKNKKAQARVIEEIDDYFKRHEGKIEYECINEMPFVQACID
ETLRLYPVLGLLTREVIEEYTLPTGLRLEKNSRVHIPVHHLHHNPEHFPEPEEFR
PERFYGDERKNVKQYTYMPFGEGPRICIGLRFSKMPMYAALLTIFKNYSVDLA
KGMPLTVDIQPRALVAQSTCDMNIKLIPRRT
>HaCYP24
MIAYYPIFTAIAAVLYFLYYNVVKYNDYWKKRNVPHLKPSLLFGNYKEYILFQ
KCLPKVARDVCRKFPNEPYVGVYYGTDPALIIKDPDIIKLVMAKDFYYFHKRE
VSEYTHKELITQNMFFNSGDTWKVLRQNLTSLFSSSKMKNMFYLIESCARSLE
NVLKQEMEKNDTIEMKGLLARYTMDCIGSCAFGIETGTLAKKSLKNPFTIMG
EKLFDVSNYGGFRMVSRALWPAIFYKLGFTMFDRDITHFFKKLLTDVFESRQY
SESSRNDFVDLVLTWKKRNYLTGDSISNIKTGDRETISLDVNDDLLISQCVLFF
AAGFETTATTTSFILYELAKNKKAQERVIEEIDDYFKRHEGKIEYECINEMPFV
QACLDETLRLYPVLGVLTREVAEEYTLPTGLKLDKGTRIHIPVYGMHHNPDYF
PEPEKFRPERFYGDEKKNIKPFTYMPFGEGPRICIGLRFAKMPITAGLLTVFKNY
RVELAEDMPLEVDFQPRALVIQAISGIYLKLIPR
>HaCYP25

MNAVIEELLSLIAEYWKLLLIINLIFYFYFYQTQTFDYFKKRNIKFKKPIIFFGNT
LSRFTSKKPFHIFQINVYNYFKGERMGGFFEGHRPRLYILDPDLIKAITISDSDH
FIDRSVVKTREPRYLSRSLLALQGGEWKAVRSLITPTFSSSRLKNMFPLIQHSC
NQLVELITSLDESEIELKNVTGHLTLEVTGVCAFGISTDGLKDKNAEFYKIAEN
FNYMSVRKRISLLFIFLFMPSLLKYINISFLNGESISKLIKILQKTKAERMSAESK
RSDFLQLLVNVALQEKVETANTTTSTKRHLDDDTLDAQALLFLLAGFETTSTL
LSFFFHTMAVQPDIQEKLRVHIEEVTQGQELTYDHLAQFEYLEATIFETLRMYP
PLARLDRACTKPYTIPGTSVHLGVGDVVVIPAYGIHMDPDIYPEPEVFKPERFM
KEERKERPSHLFLAFGAGPRNCIGLRFAMVVAKTAIVTLMRNFKFSAGPKTEN
PIQFHRSSFLLKPQNGIWVKVEKI
>HaCYP26
MIALTLFLVTLFVLYIYSKRNHTFWKLKGVTHDRPIPFFGNNLRNFLMRKSVT
EIAVEMYRKYPTEKAVGFYRASLPELILRDPAMIKRVLITDFLYFYARGLNMDK
HHIEPLLRNLFFADGDVWRLLRQRMTPAFTSGKLKAMFPLIVERAERLQARLL
TAAANEEEIDARDIMARYTTDFIGACGFGLDSDSLQDENSPFRKLGSTIFNKSP
RDVLVITLKHIFPGLFKDLKIFGQAEKQIIRLVKEVLRQRNFEPSGRNDFIDLLIE
IKKKGKIVGQSLERMKPNGDPEIASLEMDDDLMAAQVFVFFAAGFETSSSVSS
ITLHELAYNPKIQLKVQKEIDTVLAKYDNKLSYDAIKEMTYLEWTLKEGMRV
FPSLGILVRECVRKYTFDEINLTIDKGVRIIIPLQALHNDPKYFPDPDEFRPERFD
PANFDVTNKHVYLPFGDGPRACIGERLGLMQALAGLVAVLARFSVQPGPSTQ
RQPVVNPGPTSVQTIKGGLPLLFIERKSHI
>HaCYP27
MFLIFLCILIITLYFYGTRTFKYWVKKGVKYDKPAIFFGSSLKQFFDNVSVSGRF
AALHRAYPNEKFVGYYEFHIPGMLIRDPELIKHILITDFRYFHSRGLNPHKTVIE
PLMKNLFTVDGDVWKLMRQKLTPVFSSGKLKAMFPLIVERTLKLEVLAKRLA
ETGEEFDIRELMARYTTDFIGACGFGIDSAALEEENSDFRKLGRRIFRVTIRDQL
VRILKILAPETFRNLHFFPPEVERNTLSIIKQIMSERNFKPSGRNDFIDMMLELK
QKGKIVGESVEKRNPDGTPQTVELELDDQLIAAQVIVFFAAGFETSSSASSFLL
HLLAFHPEIQERCQKEVDEVLKKYDGKLCFEAVKDMKYLEMAFKESLRCLPS
PGYLIRKTVSKYTLPGTNVTLDEDVFVVISTEALCSDEQYFENPEAFIPERFHP
DNIDKIKKWTFMPFGDGPRSCIGERMGIMQSMAGVATILSKFTVEPSRNTIRKP
RIDPSSLLVEIIVGGLPLAIKHRQKI
>HaCYP28
MFLIIVGILLIVLYFYGTRNFKYWEKKGVKFEKPLVLVGSNLKQFIDNVSVSER
FAALHRAYPNEQFVGFFEANNPGILIRDPELIKHILITDFRHFCFRGLNPHKTVI
EPLMKNLFTADGDVWKLMRQKLTPTFSSGKLKAMFPLIVERALKLEVLAERL
AETGEEFDIRELMARYTTDFIGACGFGIDSAALEDENSQFRKLGKRIFRITKRD
QLVNLLKRSAPETFKNLHFFSPEIEKNTISIIQQIMSQRNFKPSGRNDFIDTMLEL
KQKGKIIGESMEKRNPDGSPQTVELELDDQLLAAQVFAFFAAGFETSSSASSFL
LHLLAFHPEIQERCQKEVDEILQKYDGKLCFEAVKDMKYLEMAFKESLRCLPS
PGFLIRKTVSKYTLPGTNVTLDKDIIVIISTEALSTDEQLFEDPESFIPERFHPDN
VEKIKKCTYMPFGDGPRSCIGERMGIMQSMAGVATILNKFTVVPSHNTVRKPR
IDPSSLLVQIIDGGLPLAVKRRQKK
>HaCYP29

MQHLRRSAFSLLRVNKQFTRSVALNNVAEKTSEDNLKSWLEIPGPSSLPIIGQM
HHFLPGGLLSYTDELLIDVLYREFGPIVRLDGYFGGPSTILLYDGDAIAQVLRS
ENWLPARPGFQSLTYYRENIFKKKSDPPDAPTGLITDHGEVWKKFRSMVNPIM
LQPKTIRLYSGILNKVAEDMVKRMRLIRNEKNMLNGNFDMEMNLWALESIGV
VALGGRLNCLDSDLPDDSPAKKLIQLVHDIFISADELDFKPSLWRYFSTPAFKR
AMKHYDDQLKISKFFIDKAIEELKTKGTSSNEEKGILEKLLEIDENVAVIMATD
MLFAGVDTAANTMTATLYYLANNPEKQNKLREEILLKQEKQQYLKACLKEA
MRLMPVVAGNMRLTSKEYNILGYKIPKNSYVSFIHQTLSVLEQHYPRAKEYIP
ERWIVEKSDPLYHGNAHPFAFSPFGFGVRSCIGRRIAELEMETFLAKVIENFHV
EWFGPPLKTKQSSLNYIVGPFNFVFKDVK
>HaCYP30
MLIVYPLILLALFCYFLYYYFTRTFDYWKSRKVPGPQPLPIFGNLKDVVFRKQ
NTVTIYKEFYDEYPDEKMVGIYRMTTPCLLIRDLDIIKHILIKDFNYFIDRGIEFS
KKGLGANLFHANEEIWRPLRSRFSPLFTSGKLKHMVYLMVERSDIFIKYVKAL
TDIQPEQNVYSIIQKYTISSISSCAFGLDIDIENKKFMTTIDKIDKLIFTRNLMQE
FDLMYPGVLKSLNLSLFPTEIVFFFKDLVDKVLQARNYKPTNRQDFIDLILELR
QQNNVLLNKKTEVGDEEFFEVTDDIITAQAFVFFAAGYETTATTMLYMVYEL
AKNPDVQEKIIAEIDETLKKYKGEITYETLCDLHYMEKTFDETLRKYPIVEPLQ
RVAKFDYTIPGTNVTVKKGQIVVLSVMGIHWDEKYYPNPKKFDPDRFSPENV
MNRHSCAYIPFGQGPRNCIGMRFAKIQSRIGLLKLFSKFRVTPSKNTPESMIFNP
MRITLSPQPNLLLNLIPRDDIK
>HaCYP31
MDLSTDSQTSTFKITLIGVLVTFSILICLYIRKIYNYWKDRGIPYDKPLPIVGNLG
FLMRRSVWDYCYELKNRHPRDYLGIFLAWTPVLVVQTPELARRILTKDFEYF
QDRYLYSGYSDPLGALNLFTIKNPLWKTLRYELSPMFTASRLKKVTELMNVN
ATELVHKVQRDINSKKDFNLKELFSMYTSDTVANTVFGIRVSILNDKPSPLWFI
TRNMVQWTFWRGLEFTMIFFVPAVAAFLRLKFFSGAATDYIKKLFWTVAESR
QKTETSNEKDLVNLLLKLREKLKLPTEPDSPLVDDVILAQAAVFILGSIETSSTT
ISYLLHELAYHPEEQEKLFNEISEAVKRKGNDVLEYNDLLELKYLTACINETLR
KYPPVPYLDRLCKNNYKLDDNFIIEKGTPVFLNVVAIHYNEKYFPEPEKWRPD
RFITLAESDNADFTFLPFGDGPRFCIGKRYGMMQVRASVAQLIQKFKMEPAVP
YAVKPDPYAVILAPENGLSVKFVPR
>HaCYP32
MQSLRRSTLYFQINNQFVRSITINNTAEPTSEQSVKIKSWREIPGPSSLPIIGQLH
HFLPGGSLSSLSVLQPGNLYKTYGPIIRLDGFFGSPGLVLLFDPEASSQILRGEN
WLPYRMGFESLDYFRKEYKNNVTDNEPTGLVTDQGEVWKKFRSTVNPIMLQ
PKTIKLYKNSLNEVAEDMIKRMRLIRNSENMLEGKFDEEMNLWALESIGVVAL
GGRINCLDLNLPEDSPAKKLIHTIHGIFKTAEEIDFKPSLWRYISTPGFKRAMKL
YEDQVELSKFFIGKAIQKLEENDVSSKEKGVLEKLLEIDEKVAVIMASDMLFA
GVDTASNTVTATLYLLAQNPEKQNKLREEVISQAEKRPYLKACIKEGMRMLP
VVSGNMRKTTKDYDILGYRIPKNTAVTFQHQFLSSMEEQFPRAKEYIPERWIT
EKTDPLHHGNAHPFAFNPFGFGARSCIGRRIAELEIETFLGKLIENFHVEWFGPP
LKIKPSTLNYTVAPFNFVFKDVK
>HaCYP33

MFKYTSFLNLNKICRVISLRSISTSNSIKSIEGGSVKDWRDIPGPSSLPIIGPLLHF
LPGGLLHDRNELQETLYKNYGPIVKIDGNFGSSTLIFIYDPEAAFHIFRNENWM
PVRPGFPSLEYFKKHYNRKKDEPCTEFTGLLTEHGEIWRKYRSIVNPVMLQPK
TVQLYKNILKEVGEDMVKRMKSRLNNNNMIDGQFDKEIYLWALEAIGVVAFG
SRLHCFDSNLPPDSPVSKLIQVVHDVMNSAQTLDFKPSLWRYISTPTFKQAMK
HYEDQIKLNEYFINKAIEHLEMKQKTNDEKGVLEKLLEIDHKVAVTMASEML
FAGIDTTANSVISLLYLLAKNPEKQIKLRDEVISKKERQSYARGCIKETMRLMP
VVGGNFRQTTKEYNVLGYKIPKDSFVIIGNQSMSIMEEQFPQPKEFIPERWIVD
KNHPLYYGNAHPFAYSPFGFGVRSCIGRRIAELEIETFITNVIENFHIEWFGSPLK
THATTINYCIGPYNFVFKDVK
>HaCYP34
MLTLILSILLTLCLLHYLFYCNNFAKLCLKVPGPPIKFLLGNSLETLVSPVELFAL
TRHWASLYNGIFRFYAYFHASIIIYNPEDIEIVTSSMKYHKKSGVYNLLAPWLR
NGLLLSSGSKWQQRRKILTSAFHFNILQKYHVALEDNSQRLIKVLEETNGEST
NIVPFISEYTLNTICETAMDTQLNEESSEAGKLYKKAIHELADLLIQRVSNILLH
PKAIFDLTSIGRKQHRHLSIIHRFTKSVIEERKKIYDDKCEGEFKLRENERNISSR
KKRKRHAMLDLLISAEKEGLIDAVGIQEEVDTFMFEGHDTTASGLIYSLLSFAN
HQDIQDKIVEEQNNIFGEDTRPATMDDLAQMRYLDCCIKESLRLYPPVPFISRQI
SEDTVLSGYKIPAGAYCHILIYDLHRQEHLFKDALKFDPDRFLPENCVGRHNY
AYLPFSAGPRNCIGQKFAMMEMKSALSAILRNYKLIPVTKHSDLRFRSDLVLR
NSGPVYVKFVKRNLVK
>HaCYP35
MKHFSGYRFTSKNHIKPFDAIPGLSSLPFLGPIHHFIPGIGMKSFLSYQKMILMI
TNETLLIIAGSVGLHANFYDLSKVLFEKFGSIVKLDGIFARASMVILYEPEHFDQ
VYRSEDTLPSRPGFDSLVYYRQVMRKNVTGGVYGLTIAEGSQWRDFRTKVNP
ALLKPKLVKLYTPALEVIAEDMVVRLIKLQEKENYLEQNLDFEMTKWSLESV
AVVALGTRLGCFDDKLTDDHPARILMKCSKDLMELAWKLEFSPSLWRYYETR
NFKKMVKTLDSQWEASVKFINETKTKINERGHDIPEEDKSVIEKLLAVDDKVA
IMMANEMLFAGIDTVSFTTICLLYNLATNQNAQEKLRNEIRSQENSNRYLRAC
LKESLRLYAVIPANLRRTTKEHTIDVVAPNEFLSRMDKYYPRAKEFLPERWLVE
KSDPLYYGNCHPMVTLPFGFGVRSCIGRRIAEMEIEVFIKRLLRDVKITWEGPP
VQVVTRVMNSLKKPYRFKFQLIK
>HaCYP36
MLYSTPPPLVDWSGVPTLVLALVALVMAATALLTRSMEGKRPSRLPGPPALPLL
GTRWLFWSRYKMNKLHEAYEDMFRRYGLVFAETTPGGAIVVSIAERTALEAV
LRTPAKRPYRPPTEIVQVYRRSRPDRYASTGLVNEQGEKWHHLRRNLTTELTSP
HTIQGFIPELNGICDDFLNLLQSCRRPDGFVHGFDQLTNRMGLESVCGLMLGT
RLGFLERWMSGRATALAAAVKAHFRAQRDSYYGAPLWKFAPTSLYKTFVRSE
ETIHLIVSELMEEARARTRGAAQDDGMQEIFLKILANPELDMRDKKAAVIDFIT
AGIETLANSLVFLLYLLSGRADWQQRIRSELPSCGELRIEDLSSAPSVRAAVNE
AFRLLPTAPFLARLLDTPMTIGGYRLPAGTFVLAHTGAACRREENFWRASEYL
PERWIDIREPHAPGIVAPFGRGRRMCPGKRFVELELHLILAKILQNWRVEFDGE
LDIQFDFLLSPKSPASLRLVEW
>HaCYP37

MIAFITVICLLLVILTSWIILIRDSKRFNVPGPLPLPLIGNGLLFVAKPSEFLPILHK
QKENFGDAFRIHLFHTPYIVLSHPRYVEALVSDVDLITKGHSYYFLRPWLGDG
LLTSTGNKWKVTRKFLTPAFHFNILQNFLPVFLKNEKILIKKLQNYIDGTAFDIF
PIIALTALDNVTESIMGVSINAQNNSESKYVKSIESLAKIIALRMRNPFVGDDAL
FNLLPYKKIQDEALDVLHSQTRAVIEMRREELRKLNITDLCGKTDIGVKNKNA
FLDLLLLSEIDGKKIDDDRVREEVDTFMFEGHDTTTSGICFALYCLSKHPEAQE
KILEEQKRILGENFDRDPLYTEVQQMKYLELVIKESLRLYPSVPLIERLMIKDTV
IAGLNIRKKSSVLINIFEMQRHPDLYDNPLEFRPERFESASANSAKNAFSWLAF
SAGPRNCIGQKFAMIEMKVTIASIVKHFVVQSGDNETLGLCAELILRSENGVK
LKLKPRIMN
>HaCYP38
MLYVLLFFVLVILLLHYYVNILNKDVNFEKIPGPKGIFLFQNGFDFLQESPALFT
YFRHYSSKYKDIYKLKLLHKKFLLILNPEDVETIISSTKYNDKGFMYYFLKPW
LNDGLLTSSGTKWHQRRKILTPAFHFNILRHFNTILVENSEKLVKNLQVEVDKP
KTNIYNYVTTMTLHSICETAMGTALDNETGIGKSYKDAIHVLGTYLLYRAQRF
WLHPMSLFNLSNVGRNQKKLLNKISSFRDHVVKQRRENGNYKKIFNEVMND
EEHDSLVDKKRRLAMLDLLLEKEEEGKIDVEGINEEVDTFMFEGHDTTATALQ
FAFMLLANHPKYQDKILEECQNIFGSSDRKPTMNDLAEMKYLECCIKETLRLY
PPVYFIIRNCQQDVKLKDYECSAGVDCSILIYDLHRRSDQFKEPLKFRPERFME
EPTWHRFAYIPFSAGPRNCIGQNFAMMEMKLAISAVVRKYRLLPITTPQDIVFI
VDIILRPKDPIFVKFEKRE
>HaCYP39
MLLTLLVVISIIGLLLYLDTIKPKKFPPGPKWLPILGSALEVNKIRQKTKYLYKS
FKELSKLYSKDGHLLGLKIGKDRIVMVNTVEANKEMLYNEDIDGRPQGIFYQT
RTWGERKGVLLTDGELWKEQRKFLIKHLKEFGFGRKGMSEIAFAEAGHMVN
DVLEILDNKDSAVVPMHNFFSTYILNTLWTMMAGIRYKPSDPQMILLQAILFD
LFSAIDMVGCPFSHFPILSVLAPKSSGYSDFIRIHQRIWQFLRDEITVHKMRFDP
NNEDKDFMDVYIRILRDNGEINTYSEAQLVATCLDMFMAGTETTNKSMSFCFS
YLVREQNVQKKAQEEIDRVVGKDRFPCLDDRSNMPYNEAIVHECIRHFMGRT
FGVPHRALRNTTLAGYNIPKETMVVSNFPNILMDEELFSEPYSFKPDRFIVDG
KLCLPDYFFPFGLSKHRCMGDILAKCNIFVFTTTMLQRFSLLPLPGGPPPSLDH
VDGATASAAPFDALVVRRI
>HaCYP40
MFILILICVLFGVMTWFGKRRLKNNPPALPGALPLIGHAHLLYRNREPFRLWN
LFKEWSYECLKQDGLVTVYFGPQTIYALSDPDDFLTVANACVQKDNIYEFGKP
WVGEGLLTANSSTWKIHRKLLNPAFNPIVLDGFLDVFNKQSRRLVKKLEIEIGK
ESFDPISHVKNNALETVCLTALGLDLNEKSELNSQYIHSVYQACHIFMERSKRF
WLHNDFIYSWSLLKKQQDECVKIFHTMSKTILQRTKADYVYNNCQTEEKPQG
PKFMAFMKLLLELTIEKKLLNDDEIREEVDTIIVAGYETSAIVLIFCFIMIGSYPK
VQEKVVNELHEVFGEDDRDVTKHDLSRLVYLDAVIKETLRVYPIIPILARYLD
KDVKLRNYMLPKGARCFVSLYGIHRSSVWGSDAEEFKPDRWMNPASLPKSST
AFVAFSSGKRNCIGKSYALMSIKTTLVHFLRNYKVQGDHTKMILELDIALKPV
SGHHISIKKINNKNRI
>HaCYP41

MLFVLLIFVCVILSLIFYEKYRKNYWKKHGIVQVDGILSKFTWGNRSIAEVYK
DVYDDHPTESCIGMYLGTQPALIVKDVQDIQAVLQGNFENFHSRGIFSNPKDIL
SDNVLFMGDYRRWKLLRNKLSPVFTSMKIKNMFYIMERCAQDFVKFLDSEQ
FTPDNTFNALYTYTTACIGATIFGIDTHTRNTMDSPFLEMTRKSIEPSLINNIKFS
LANMSPTLCNLLNLKRFGDSEEFFIGTVKRVLNIRRNTEEKRHDFVDMCLELQ
RQGTMRDTVTGYEIEATDEVLAAQAFFFFLAGVDTSATVMHFTLLELASNQNI
LEKLHMEIDRVFDECDEKLTYEDISKLEYLDMVMSESMRMYPPIGSIQRCCTK
NTYLPTSKVQVKENDFIIIPVFALHRDEKYYKNPNIFDPERFTTANTSNIVKFSY
LPFGEGNRMCLGTRFARVQVKSGLAWLLRRFTLKERKYEPKTFAPSFFSLRDT
KSNFELIPRKKSC
>HaCYP42
MLILMLTCLVLGLLVLNRFIQRKPNKDEPPVLPRALPFIGHAYLWLGSTTRFW
NTLKKWSYDSQNHDGVASFRIGPMTLYVISDPDDFLTVCNACLDKNEFYDFA
KPWLGEGLITGKASIWKIHRKMINPSFNQIALDGFLEIFNKQSRHLVKNLEIGIS
KTSFDSYTYIQNNALETICQTALGLDINDKNMRNSQYLLAVDEILNVMMKRIQ
KLWLHSESIYKWSSLKKKQDECLKILHTTSNTILQKTKAEYLSNKSSEYNQGP
KFRTIMRLLMELSVEEGAFNDREIRAHVDTMIAAGYETAATALMYCILMIGSY
PKVQEKVFEELHDVFGDDDRDVTKEDLSRLYYLESVVKETLRVYPVVPFVTR
HLDKDVKLRNCTLSKGATCLLSIYGVHRSTKWGPDAEEFKPERWLNPASSSK
SPAPFAGFSVGRRSCIGKTYAIISLKTTLAHVFRNYEIKADHKKMEMKFEIVLK
PVSGHHIFIKRRTTQN
>HaCYP43
MLWQILLPILVCVVIWKLFKTEDNDLYRLPGPPAWPIVGSALSFLGLSHVQMF
ELLLEFPKKYGNRVVFRVLNRLILHIYNVEDIEIVLSHSRNITKNKPYSFIEPWL
GTGLLLSTGPKWHSRRKILTPTFHFDILKGFMRVFEEQSRNVVTELRRMTSVG
SGVVDVMPFVSDFTLYTICETAMGIQLGADKSEAKLKYKDAIMDIGQLVMKR
LTTIWLHSNFIFSMHPMGKKFAQCLNNVHSFADSVIMERKQAYENDGEVLAG
DSGSKRRLALLDLLLEAERKGDIDLEGIREEVNTFMFEGHDTTATALTFGLML
LADHEDVQKRIFEECKSVLGDTDRSPNASELAEMKYLEAVIKEILRLYPSVPFI
GRTIVEDFMLGDIKVKKGSEVVVHIYDVHRKPDLYPEPDAFKPERFLEGDSRH
PYAYVPFSAGPRNCIGQRFAKLEMKSVISEIIRHFKLEPLQRGARPTLKSDLVLR
PNEPIYVKFIQR
>HaCYP44
MMRIILQLEHFSDDIERLYTTFPEERFIGKYEFIKPTVMINDLELVKKITIKDFEH
FLDHRVVTDENVEPLFARNLISLKGQEWKDMRSTLSPAFTSSKIKLMVPFMEE
VGEQMIRALKKKIKESDTGYIEVECKDLTSRYANDVIASCAFGLKVDSHTDEN
NQFYKMGKEISTFTVKQIFLFVIFSAFPSLAKRSNLTIFSRKTKNFFVGLVLGTM
REREDRNIIRPDMIHLLMEAKKGKLTHDNLGNNDKDTGFSTVEESSVGKTTID
REWSDMDLVAQAVLFFLAGFDTISTAMSFALSELALHPEIQERLAQEIKEHHVK
NGGKLNFTSIQNMTYMDMVTSEILRLWPPAIALDRVCVKDYNLGKPNSQTSK
DYYIRKGEIISIPVWCFHRDPAFFPDPKRFDPERFSDENKHKINPMAYMPFGVG
PRNCIGSRFALCELKTLLYQILLHMEISPSEKNCLPVKLCTESFSPRMKGGHWV
KFKART
>HaCYP45

MWLVYLAVFILVLVIVDRWKSKEMVNLHKELDVGITYVPVLGHVYKLIGNGE
VRMAVLEEVGRKALNNVHRMASMWIFNYTWVVVSDPEAANFILKTCLDKG
RLRSFARHLFGNGSIFASVNVWRPRRKVLAPMFSLKKLNKFIEIFEKNSIVMVE
QLASVVGKGNFSIEKFINTYSFDTSCETTLGESVNSQRQADHPFLVALTNYAEN
IIDRMCKPWLHIRAVYKLTESYADQVKYKKTIYNVVEKVIKKLYCRLLSTQVQ
SFLETLTDLSRFNDVELREEALILLLAATETTALSSGYTCVLLAHHADVQDRVY
KEIQDVLGDSDRSICADDLINLKYLDAVLRECLRLYPPAPMLVRTCHADVTLPS
GLILPKGTNTVINTWVIHRNPQYWGQDANEFRPERFLNVQRDQLASFLPFSSG
PRNCLGYHYAFLTMKTNLATLLRRYRIVPATSFKYDGRSPLRVKFSATLKHVH
DYEVQLESRV
>HaCYP46
MYSLISICIVLCLLMWFSRKRNKNEPPALPGALPLIGHAHLLFRNSTEFWNLLK
DLSYKSQKADGIISLQIGPQTVYVLSDPEDFLTVSNACLQKNVSYDVPKTWIG
EGLIVAKSSIWKVHRKLLNPAFSQKILDGFLDVFNKRSREFVKLLDIEIGKRSF
DPYNYIRHHALETICQTTFGMDFNDGQFIMAVDQICHIFAERVQKLWLHNFMF
SWSSLKKKQDECLKILHSMSNTMLQKMKIDYSNSKNSMENTEGPKFKALLN
YLIELEAERGVLNDQEIREEVDGTILAGFETSATALLYSMLTISSYPEVQEKVIE
ELHEVFGDDDRDVTKHDLSRLYYLDAVLKETLRIYPVIPGIARHLDKDVKLRN
CTLSKGSTCFMLIYGVHRSPIWGPDAEKFYPERWLDPTSLPKLPTAFAGFSAG
RRNCIGKTYAFMSVKTTLVHIFRNYKVKGDHTNMQLKFDVVLKPVTGHHISI
QRRNKILL
>HaCYP47
MYAIAITILVLIITGYIIKSTRKPKNFPPGPKWYPIFGCSNLVHSMARKQGSQWK
SLSMLAKEYKTKVLGIKLGPEPIVVTFGENNVRRVFTEKEFEGRPTSFFIRLRC
LGKKMGITFADGTLWRIHRQFTVKHLKNVGFGKTVMESEIQKEMQNILNYIA
DNGNKPINPKNILATSVMNILWKFTAGERIKGDRLNFLLDLLNTRSKAFSMAG
GWLNQWPWIRFFIPEISGYTLIKNLNQQISDVIEEAITKHKQNLILENDFMYSFL
EEMKENKETFTEEQLKIICLDILIAGSQTTSNVLEFAILKVMKDKIIQEKIFDEIS
KILGDDLPSWNDTSRLIYTMAYLYEIQRFFTIVPLAGPRRALDDINMDGYLIPK
DTTILISVGDVHFDPEIWEEPDKFMPERFIDKTGRITNIEHIYPFGIGRRRCPGDS
LAKSFIFIVFVGIMQRYRIECTNGIVPSEEPIIGLISSARPYSAVFIPRP
>HaCYP48
MLYYLFVILFILLVVDRWKPRNLVKLHQKLGTDWQYVPIIGHAYKFIGNNEGN
NVSLFSLILLKCYDSWLIYIVVYPINLFCLVTADIIEATSVLKTAMDKSFIGRFTY
NLIGNGSVHAPAEIWHRRRKILVPSFASRHIKKYLRVFEANSKMVAKQLGSRV
GKGNFSSWNYICRQTLDSICETTLGVNIGVLKEQDHPFFKALEEVGKIIAVRIC
KPWLQIDALYKFLPIYKRQKYTTKIIHEFIDNVSTTFKSFLELMIENSEKTDTGY
TQEELREEALVLLIAGTDTTATAICFIFVLLSQHQHVQEKVYKELEEILGDTNKP
IEVEDLVKLKYMEAVIKETLRLYSPVPVLTRDSKNDFQLPSGLTVPKGCDVIIHI
LGIHHNPRYWGADVEDFKPERFLKDEKPDAFIAFSNGPRNCVGFLYAMISIKA
TLSIILRRYRLLPATSFRYNKKNPLRLSFDIFTKHLDNFEIQIEYRN
>HaCYP49
MWLVCLFSIVLLLVIVDRWKSKEMINLHKELATDITYVPVLGHVHKLIGNSEE
RMAAVQEIGRKSFNNRHNMASVWIFNYMWVVISDAEAANIVFKSCLDKGRLI

SFARHLFGNGSIFAPVEIWRPRRKLMAPLFGLKTLNKFIKIFEKNSLVMVEELA
SAVGKGNFSIEKYINAYTFDTSCETILGESVNSQRQTDHPFLVAFSNYTENLIDR
MCKPWLHLGAVYKQTAAYIDQVKYKETISAVITGKSHIQTFLGSLTEHSGYND
VELREEALILLLAATETTALSSGYTCVLLAHHADVQDRVYQEIRDVLGDSSQA
ICSDDLNNLKYLDAVVRESLRLYPPAPMTVRACHSDVRLPSGIILPKGTNTVIN
TWAIHRNPQYWGKDADEFKPERFLSAQREQLTAFLPFSSGPRNCLGYHYAFLT
MKTNLATLLRRYRILPATSFKYDDQNPLRVKYCATLRHVHDYEVQLENRY
>HaCYP50
MQEIFNAKSVTDMAVEMYWKYPSEKVVGFYRGSRPELIIRDPEIAKRILTTDF
AHFYPRGLNPHDQEIEPLLRNLFFADGDLWRLLRQRMTPAFTSGKLKAMFPLI
VERAERLQVRALSAAAAGQEIDARDLMARYTTDFIGACGFGLDSDSLKDENS
AFRQLGAKIFNFGAKEILIVALKEIFPFLFKNLKTLTRVDKDMYRLVNEVLRQR
NYEPSGRNDFIDLLLECRKKGTIVGESIERTKSDGKPEVATLEITDDIIAAQVFV
FFAAGFETSSSATSMTLHELAHNVEVQNKVQEEIDRVLAKYDNKLCYDAIKE
MRYLEWAFKEGMRIFPSLGFLIRGCARKYTFQDLDLTIDENVRVIIPLQAMHN
DKKYFENPTEFRPERFDPENFDADNKYVYLPFGVGPRACIGERLGLMQSLAG
LAAVLSRFSVRPARSTRRRPAANPASGIVQTVSGGLPLLFIERTNSVS
>HaCYP51
MFYYLLLTIILLLLVDLWKRWKYLELNRAIGIDCIYVPFIGQAYKLFGDNETIYT
IMKKAISYGYNRKHGTVGGWFGNELVVGTVDPIEATAVLKTAFNKTFIYEFAF
ELLGNSTVFAPATTWHRRRKILASTFAPRLVNKFIRIFEKKAKLLVQLLEPKVG
RGNFSCWDLISSYELDSVCETTLGVDINVLRDPDQPFVKAYEEIFKTALTRLFR
PWLQIDVLYKLLPVFRRQQHRKQIIYEFIDNIIYKKEEMLREKAINKDKIDNGT
NNISFLELMIEGSEEAGKRYTPEELREEAVMLLLAGTDTTSTGLCFIMVMLSQ
HQDVQENVYKELKEVLVSAIVRDSEQEFVLPSGVTIPKRCNVITSIAGINCNPH
YWGTDAHIFKPERFLSKQTLVPGAFMSFSYGPRNCIGYSYAIMSMKTVITTILR
QYRLLPATSFKYDENTPLRLSYEFMTKHVDNYEIQLEYRH
>HaCYP52
MSGPLPIIGHTHLLLGNTTQLWTFLKNLYSEIQKQNVTCALHLGPIKVFYVVSN
PDDFLTVANTCLEKAKFYEFAKPLAGEGLATGQVPIWKFHRKLLNPAFNQIVL
GGFLDVFNKQARRLVNDLEIEIGKSSFDHYRYIKHNALETLCLTALGVDLKEK
SELNRQYIMAIDVIANLLIERLQKPWLHINFMYKWSTLKKRLDEVSKVVNTM
CETILQKTKVNYLMNKNREVEHMQGSKKTFMYLLTELSIEKGVLNDREIRDE
ANTMIAGGYETSATALMFCVLMIGSYPKVQEKIVEELNKVYGDDDKDVTKY
DLTRLIYLEAVIKETLRLYPIVPVVARELEKDVELSDCTLSKGGTCLLFIYGVHH
SSIWGPDAEEFKPERWLDPDSLPKSATAFVGFSAGKRNCIGKGYALMFLKTSL
AHLFRNYKVKGDHKKMELKLDITLKPVSGHHISIQRRNI
>HaCYP53
MKKSAPEITSEICDQFPDEPYVGILYGTEPALIIKDPNIIRLVLSKDFYYFSGREIT
SYAHRETITNNLFFNGGDEWKVLRQNLTPLFTSAKLKKMFPLIQTCNNELEIFL
KEETRISETIHARSFFARYAMECIINCAFGINANTMKRNIDNNIFFIVAQKIFDST
FMRGLKMACRAMWPFLFYGLRFELFDEKIVTFFRTIFTEVSKNRANEKSTRN
DFVDLILNWNEQKYITGDSLSNMKTGGNKVNSIEVDEELLLSQSILIFAAGFET
TSTATSFLLYELSKNKKALDKVIEEVDTYFEKHSVIEYECMHELPYIEACIDEA

LRLYPVLGVVTREVMDDYVLPTGLRLKKGDRIHIPVQHLHKNPDYFEDPEMF
RPERFLGDEKAKIKPFTFLPFGEGPRTCIGIRFAKMVMFPQFLTLFKNYRIELAE
GTPLSVEFNPASIATQSMTDLKIKCIAR
>HaCYP54
MTRKHGSQWKTLSVLAKEYKTNVLGLKLGSEPVVIFFGENNVRSAFKEAAF
DGRPESFLRKGMGITFADGTLWKVHRQFTFTHLKAVGFGKPMMESEIQKETR
AILSYINKNIDKPIDPKNMLAAPIVNILWKFIAVELDAKREHRRSAFSFAAWAN
ESSSLRRGGKDDLQKFPRIDLARDQNARVPERRRTSLSPLLPMFSDFAFPGGRI
VLLSNYADLEDAIKKHKKNLINENDFMYSFLKEMKHNIATFTEEQLKVTCLDI
IIGGSQPSSTVIEFAILKVMWDKNIQEKIYNEIVNNLGDKLPSWSDSDRLVYTK
AYLYEIIRYFNIAPLAGPRRALHEVNMGGYVIPKDTTVLMSIGDVHIETDIWDE
PDKFMPERFIDKTGCLRNIEHLYTFGLGHRRCPGDTLSKSFIFIVFTGIIQRYRIE
CVNGILPSEEPIISLLSHARPYEAQFITRH
>HaCYP55
MGIFWDFLTTNQAMFEHLHDIYKQYPNDPAVGLGSMLTPTLYVRDPANVQFV
LSSEFTSFSHRGFEANEGDVLAENILFLNGRKWKLMRQSMTPLFTAAKLRNM
YYIMDKSAQDFVGYLKESPKLMKGDTFNNLSTFCCAAISAAVFGVTTESIFNS
PFLDVAKKAFQSELMRDIKFTIGNLSIRLFKILKLKVFKEYEQFFVGAIKKVVR
LREKENVKKHDFADICIALQSKGKLKDSESDFELEPTDELLAAQGFFFFVAGV
EPTAAAIFATLVELGKNPYILQRVHEEIDNIFNNCDGKLTYDIVANINYLDMVM
SEALRLHPPIGFLTRMCVKEAVLPTGNIKIDKGTKIITPIFEMHHDAQYYPNPEV
FDPERFSRENRNAVPDITYMPFGKGNRICIGMRYAQLQAKAGLVHLLRNFTVK
THVSKGGIKYRKDQVQVRLINVDVEFISRN
>HaCYP56
MLIILLSCLVFGVLLWFQRKRNKNEPPVMSGTLPIIGHVHLLIGNITNFWNFLEI
LVSECMKNDGVATFCLGPIKVYVPIWKIHRKLLNPAFNQIVLDGFLDVFNKQS
RRLVKGFEIEIGGEPFDHYIYTKNISLETICLTALGVDLSERSELNSQYIMAVDQI
FKIFMKRGQRFWLHSNFIYSWSALKRKQDECLKILKTMSYTILQKTKENYLN
NRNKTDEKGPQFKALMQLLLELMIEKDVLNDTEITEEVDTMIAAGYETSATA
LAYCVLMIGSYPEVQEKVFNELYEVFGDDDRDVTKHDLSRLTYLEAVIKETLR
FYPVVPVIARYLDRDVKLRNCTLSKGSRCFLFIYGVHRSSIWGSDAEEFKPER
WLDPASLPKSPTAFVGFSAGRRNCIGKGYALMFLKTTLAHLFRHYKVNGDHT
KMELKIDVTLKPVSGHHISIQRRNI
>HaCYP57
MFYYFRTLCNKYKRLFKLRVGHVKIIIIHNPEDVETVVTGTKHITKGFVYDFIQ
PWLQGGLLTSKGSKWHQRRKMLTPAFHFNVLANFKSVIEENCRSFVESLQVE
VGKPQTDITPYINDFAINSICETAMGTKLDKEASSFGKAYKEAIYKLGQFAVYR
AQRIWMHPEFIFNLTILGRKQKQILNNLTSFRDIVIEKRRVLNKGLSNGFGDEF
NNDNSEEIEVYSKKKLAMLDLLLKAEKDGVIDKQGIGEEVDTFMFGGHDTSA
NALQFTMMLLANHPDVQEKVVEECNGIFGSSDRSATMADLAQMKYLECCIK
EGLRLYPPLPVIMRKVEHPLKLGNYEVPIGAECGILIFDLHRRSDQFVEPQQFR
PERFLTEPTWHPFAYIPFSAGPRNCIGQKFAMMELKLALSAVLRRYRILPVTVP
RDIIFITDYVLRTKEHIFVKLEDRI
>HaCYP58

MFVVVLFLLFLSVYYIYFRYKRRALYELSKSLPDTGYLPIIGHTHWFIGGPERV
LNNIQQLSRLVQATGEIGKIWIGPSLYIVSLNPDDVQNILENCLQKDSSYRFLQT
WLGNGLFVAPVDLWKVHRKVLLPIFHNRIIEDYIDVFGEQGSVLVERLEEQLG
KSEFDVFKYVTSCMLDIVFETAMGEKMDVQHNPDTPYLRARSTVISIIGMRLF
KAWMQPDCLFKLTSYSKLQKENIDLTHKFTDEVVRKKKELFKKQAKNIKEGR
RDLLELLLDREMKFTDEELRDHIDSITIAGNDTTALVISYALMLLGNHAAEQER
VYLELKDIFGDSKRSPTKEDLNKMECLDRVIKETMRLYTVVPIIARKTQKEIVL
SKCTVPPGVGCAVVPFVMHRSKQIWGPDADYFIPDRFLPEVSANRHPCAFIPFS
YGSRNCIGN
>HaCYP59
MGTKRVAVLLNPEDVEILLCSTKSNHKGYYYGFFRQWLNEGLLLSDGKKWH
QRRKTLTPAFHFNILRHYNSVLIENTNNFVNQLQSEVYNTKTDIYPYLTDFSLN
SICETAMGTVLDEEASEIGKIYKNAVHKLGSYIYYRGLRIWLYPDFIFNLTPVG
RDQKRLLKLIASFRNEVIERRKKSNNYKTISTELMNEDLDDVFVYKKNRFAM
LDLLLEAEKEGTIDRAGINEEVDTFIFEGYDTTATGLQFAFLVLANHNDAQDKI
VEESNRILYSNGKCKPTINDLAQMKYLEACIKESVRLYPPVHLMSRTSNQPIQL
KNFKCPAGTDYFIPLTPLHRRSDQFIDPMEFRPERFLVEPTWHPFSYIPFSAGQR
NCIGQKFAMIEMKLVISAVLAEYRLVPVTKPEDIVISLDMMLRTEEPIYVKFEK
RNKTM
>HaCYP60
MESSVAPPIILLYDGDAIAQVLRTENWLPVRPGFQSLEYYRKSILKRESPDTPTG
LITDQGDVWKKFRSMVNPVMLEPKTVSLYRGILNKVAEDMVKRMRLTRNEK
NMLNGNFDMEMNLWALESIGVVALGDRLNCLDINLPEDSPAKKLIQLVHDIFI
SADELDFKPSLWRYFSTPTFKRAMKYYDEQLKISKFFIEKAIKELKTKQTSPDE
EKGVLEKLLEIDENVAIIMATDMLFAGVDTAANTMIATLYYLAKNQEKQNKL
REEIRLKTEKQYYLKACLKESMRIMPVVAGNMRLTTKEYNLLGYKIPTNMYV
TFVYQALSTMEKHFHRSKEFIPERWIVEKSDPLYHGNAHPFAYSPFGFGVRSCI
GRRIAELEMETFLAKVIENFQVEWFGPSLKTRPSSLNYIIGPFNFIFKDVL
>HaCYP61
MQIRAMQIRAMQIGAMQIRAMQIRAMQIGAMQIRAMQIRAMQIRATYIIIPPL
LSNTDLITKGYSYDFLRPWLGDGLLTSTGYKWKTTRKFLTPAFHFNILQHFLPI
FLKNEKILIKKLNNYTDGTPFDVFPIIALTALDNVVESIMGVSVDAQNNSESNY
VKSIESIARIISLKMRNPLVESIFNLLPYKKEQDEALDVIHSHTRKVIEIRREDLR
KSNITKLNGDSDLGIKNKQAFLDLLLLSEVDGSKIDDDRVREEVDTFMFEGHD
TTTSGISFALYCLSKHPDVQEKVLEEQKIILNNNLDRDPTYIEVQQMKYLELVI
KESLRLYPSVPLIERLMIKDTEIAGLKIRKNASVIVNIFQMQRHPDLYDDPLEFR
PERFQLATANSSKNAFNWIAFSAGPRNCIGENEIP
>HaCYP62
MQKSSIAMKATEIYNRYPNERVVGFFRSTTPELVIRDPEIVKRILITDFHHFYAR
GLHPHKKVIEPLLRNLFFVDGDLWKLIRKGFGPAFSTGKIKAMFPIITESADNL
QLLAAEITTLDYYDMRELMARYTTDFIVKKILKDRNYQHSGRYDFIDLMIELK
QKGKVTVDSLEQKDENGLPKKVELELDDSLLIAQIFVFFGAGFETSSTTASYTL
HQLAYNPDYQSKVQEEVDRVLQKYNNQITYDAINEMCTLEKAFNEAMRMYP
SVAFLMRKCTSTKYTFPEIGLTINEGVNVIIPVQAFHNDDKYFREPKKFNPDRF

DSNKNMKNNIFLPFGDGPRACVAARLGKVLAMTGVAAILHKFTVEPCSISKLS
PIPQPMATVSESFVDGLPLKLRERIKNQ
>HaCYP63
MLIIPISCLVFGVLLWYQRKRNKNEPPVIPGALPIIGHAHLLIGNITRLADPDDFF
TVANTCLQKDNFYDFAKPWIGEGLISGGGPKFKPLMQLLLELMIEKEVLSDRE
IREEVDTTIVAGYETSATTLAYSVLMIGSYPEVQEKVFNELYEVFGDDDRDVT
KHDLSRLTYLEAVIKETLRLYPVVPAIARYLDRDVKLRNCTLTKGTRCFMFIYG
VNRSSVWGSDAEEFKPERWLDPASLPTCPTAFVGFSTGRRNCIGPKFRTIMRLL
MELSVEEGAFNDREIREHVDTMIAAGYETTATALLYCILMIGSYPKVQEKVFE
EYLPTRWTDDDDDDDDDDDDGDDDDDDDADDADADADDNDDDDDDDDD
DNDDDEDPDDGDNDDDDDLITHYCFSILQF
>HaCYP64
MFVYSLIKPFVGSELVAASVPVWKRNRRIIENAFKQNILDGYNELFNEQAKRL
TFAMANKLNKEFDFSEMITRNTLESVCQTTLGINLNDNNTTTNNYLRAVNRIL
EIMTERVTYPWLFINFIYRWTSLKKEQDDNLKIISNLFDQVIKKRKAEYRDGLN
NEGKTENTQEFRSSLDVLIENSVTKDSEILSDLQLNHIINNLILAGFDTISPDLLI
TLINIGSYIEVQEAVYEEVRSVMGDNETLTKEDLKRLTFLEAVIKETLRLYPVGP
IVARSTTTDIQLQEYVLPADCHVIVHLWAVNRNKKYWGSDADEFKPERWLNE
SVPSVPSAFASFSLGRRNCLGKSYGMTYMKTILAHIIRRFKITADDKKLECDLA
VMMKPSRGHSIKLESRV
>HaCYP65
MSSRLPIIGHAHLLLGSSTVLTDPDSCLTVANTCLEKNKYYAFAKPLTGEGLITG
KASIWKVHRKLLNPAFSQTVLDGFLDVFNKQSRRFVKNLESEIGKSSFDHYTY
IKLNSLETLCRSKFKTFMHLLIELAEERGVLNDGEIRDEANTMIAAGYETSATT
LMYCTLMIGSYPKVQEKLVEELNEVFNDDYRDVTKHDLSRLTYMEAVIKETL
RVYPVVPIVTRELDQDVKLTNCTLSKGCTCLLFIYGVHHSSIWGPDADEFKPE
RWQNPASLPKSASAFIGFSVGKRNCIGKAYATMFLKTTLAHLLRNYKFKGDHT
KMELKFDVVLKPVYGHHISIQKRDNKNLI
>HaCYP66
MLILVLICLGFGLLLWYPRKRKTDEPSALPGALPIVGHAHLLLSTVPIWKIHRK
LLNPAFNQKVLDGYMDVFNKQSRRLVKELEIEIGKKSFDHYKYIQHNALETIC
HVKFKTFMHTLLEKRVFSDCEIREEVDTMIAAGYETVGTAIMYTVLMVGSYP
RVQEKVFAELNQVFHNDDRDVTKHDLSRLFYLEAVIKESLRFYPVLPIVARYL
DQDVKLRNCTLSKGCTCVLFINGVHRNPITWGPDVDEFNPERWLEPASLPKSP
GAFAGFSMGKRNCIGKAYAFMSMKTTLAHIFRNYKVQGDHTKMKLKFDIVL
KPVSGHQISIQRINDHCI
>HaCYP67
MFTSGKLKKMFPLIQSCNKELEQHLQEETSKTQVIDLRSVFSRYAMECIINCAF
GINARTMKRADSPNPFVIVGQKIFESSYTRELKMICRSMWPSLFYGLGFQLFD
EKIAIFFRNLYNEVYSNRVKEKSTRNDFIDLLLTWLKNKHLSGDSLSSMKSDD
NKVISIEVNEELLLSQSILIFGAGFETTSTTIGFLLYELAKNQNVQAKVIEEVDA
YFKKHNGIIEYECLSELRYIESCIDETLRLYPVFGVITREVTDDYVLPTGLRLHK
DDRVHIPVYHIHRNPNYFKDPEIFRPERFLGDEKMKIKQYTYMPFGEGPRICIG
N

>HaCYP68
MGTVLDEEASEIGKNYKNAVHKLGSHIYYRGLRIWLYPEFIFNLTHVGRDQKR
LLKLIASFRNEVIERRKKSNNYKTISTELMNEDLDDVFVSKKNRFAMLDLLLE
AEKEGTIDRAGINEEVDTFIFEGYDTTATGLQFAFLLLANHNDAQDKIVEESNR
ILYSNGRKPTINDLAQMKYLEAFIKESLRLYPPVHLMSRTSDQPIQLKNFKCPA
GTDYLIPLTALHRRSDQFIDPMEFRPERFLVEPTWHPFSYIPFSAGQRNCIGQKF
GMIEMKLAISAVLSEYRLLPVTKLEDIVITFDMILRTKEPIYVKFEKRNKTM
>HaCYP69
MLTLIMVSVIFGLLFTYYQRKRNKTEPPVIAGGLPIIGHTHLLLGSSTLLSDPDY
FLTVANTCLQKNKFYEFAKPFAGDGLVTGKGPKFKTFMHLLIELAAEKGVLSD
REIRDEANTMIAAGYETSATTLMFCILMIGSYPKVQEKIVRELDEVFGDDDRD
VTKHDLSRLTYMEAVIKETVRVYPVVPVVTRELDQDVKLTNCTLYKGSTCFM
FIYGMHHSSLWGPDAEEFKPERWLDPASLTKSASAFVGFSAGRRNCIVTHSSA
GMGAMAQEVCGLGAELPYPLKLQGPILRHSGTSGDHRSIPPRRPGA
>HaCYP70
MYTVMKKAISYGYHRKHGTGGGWLGNELIVGTVDLINATTVLKTALNKTVI
YKFAFELLGNNTIFAPGTNNRSFLELMIEGSEETGKRYTHEELREESLALVLAG
TDTTSTGLCFIMVMLSQHQDVQENLYKEIKEVLGEKNDPLDVSDLPKLKYMD
AVIKETLRLYPPVSVIVRDSEQEFVLPSGVTIPKGCNVITSIAGINCNPHYWGTD
AHIFKPERFLSKQTLVPGAFMSFSYGPRNCIGYSYAMMSMKTVITTILRQYRLL
PATSFKYDENTPLRLSYEFMTKHVDNYEIQLEYRH
>HaCYP71
MKDGISHGYKSIVDPKEATTVLKTAFNKTLIYEFAFELLGNSTVFAPGTNNMSF
LELMIEGSEEAGKKYTHKELREESLLLVLAATDTTSTGLCFIMVMLSQHRHVQ
ERAYKEVKEILGEKNDSIDVADLSKLRYMEAVIKETLRLYPPVSAIVRDSEKDF
VLPSGVTIPKGCDIITSIAGINRNPRYWGTDADIFKPERFLSDQTPVPGAFMTFS
YGPRNCIGYSYAMMSMKTVITTILRQYRVLPATSFKYDENTPLRLSYEFMTNH
VNNYEIQLEYRHQVNGHPH
>HaCYP72
MSDTILQKTKADHLRNTNRKAENSQGIAFVQHINSHFYNKDVTIYKICKFPGP
KFKTFIRLLIELATEKGVLSDREIRDEANTMIAAGYETSATVLMFCVLMIGSYP
MVQEKIVEELNAVFNDDDRDVTKHDLSRLTYMEAVIKETLRVYPVVPIVTREL
DRDVKLKTCSLSKGSTCLMFIYGVHHSSTWGPDAEEFKPERWLDPASLPKSA
NAFVAFSAGRRNCIGKIYALMFLKTTLAHLFRNYKVKGDHTKMELKFDVLLK
PVSGHHISVERRNNKNII
>HaCYP73
MVMTDAKQAMAVLKTSSNKSFIHNLVSDLLGNSTVFAPGFKTFLELMIESSKE
TDKGYTHEELREESLVLILAGTDTSAVGLCFTAVMLSQHQDVQDKVYKEIEEV
LGDTNRPIEFEDLLKLKYMEAVINETLRLYPPVTILLRDIAKDLILRDFVPNGCD
FLISILGIHRNPQYWGEDAGDFKPERFLSGESRVPGSFIPFSYGPRNCIGNLYGIL
SMKTTLLTILRRYRLLPATSFVYDQNNPLRLAYEIMTKHVDNFDIQIENRDQRK
VDTN
>HaCYP74
MDTQLNEESSEAGKLYKKAIHELAILVVQRLSNILLHPKAIFDLTSNGRKQRKH

LSIIHNFTKSVIEERKKIYEDNSELKLRENERNISSRNKRKRHAMLDLLISAEKE
GLIDAVGIQEEVDTFMFEGHDTTASGLIHGLLSIANHQDIQDKIVEEQNNIFGE
DTRPATMDDLAQMRYLDCCIKESLRLYPPVPFISRQISEDTVLSGYKIPAGAYC
HILIYDLHRQEHLFKDALKFDPDRFLPENCVGRHNYAYIPFSAGPRNCIGIIN
>HaCYP75
MQKEKHNQAEGDSHYDLNSYQNKNFLDILITLSGGEKGYTDLELREEILTLTM
AATDTTAVSSGYTLKLMAKYPEIQEKVYEEICEVLGDTNRPIVKEDLLKLKYL
ERVIKESLRLYPPVPFVIRKIEAEIELPSGRILPSGSGVVISIWGCHRDSKYWGPN
AEHFDPDRFLPERLNLPHPCNYMPFSNGPRNCVGYQYAFMSMKTVLATVLRN
YKVVPEPENGPIPHIKVKLNVMMKAVDGYQVALEKRNT
>HaCYP76
MVINDPSTGCDVIPSDELLAAQYFLFCLGGIDNIAVMLHFAMLELSRHQKILKR
LHNEIDGILSDENKKLTFEDLENLKYTDMVISEVLRKYPPVFAIQRRCTNDTVL
PSTQERVSKGTAVVVPVFALHRDPKNFPDPDKFDPERFSNENLSKIKSFSYIPFG
EGRRRCLGVRFGRQLSKFCLVSILRKLTLKEQDNEIKTFDPSFFTLRNTLARFE
LIPRN
>HaCYP77
MIEKEILNDNEIREEVDTMIAAGHDASATTLAYCMVMIASYAEVQKNVFNELY
EVFGDDDRDVTKHDLSRLTYLEAVIKETVRLYPVIPIIARYLDRDVKLRNYTLS
KGSRCVMLVNGVHCSPICGSDAEEFKPERWLDPDSLPKSPTAFVGFSAGRRNC
IGKAYGLMSLKTTLAHIFRNYKIQGDHTKMELKLEVTLQPVSGHHISIQRRNN
KYRI
>HaCYP78
MLEISETGENAYTLEEIREESLILMIAGTDTSATAICFTAVLKEVLGDTNRPLELE
DILKLKYLDAVVKETLRLYPPIPFIARDSARDLALPLGVTVPSGSDFIIGIAGIHR
NPQYWGADAEHFKPERFLSGITPVPGAFMPFSFGPRNCIGYLYAMLSIKTTLVT
IVRRYRLLPATSFAYDKEHPLRLSFEIITKHIDNFDIQLQHRVRSG
>HaCYP79
MELLLELTIEKEILKDDEIREEVDTMIAAGYETSATVLTFCMLMIGSYPEKVFG
DDDRDLTKKDFSNLIYLDAVIKETLRFYPVAPVVARYLDKDVKLRNCTLSKGA
RCFMFIYGVHHSSIWGSDFEDFRPERWLDPDSLPKSPTAFVGFSAGRRNCIGK
AYALMSLKTSLAHILRNYKLQGDHTKLELDLDIVLKPVSGHHISIQRRK
>HaCYP80
MIENSEKTDTGYTQEELREEALVLLIAGTDTTATAICFIFVLLAHHQDVQEKIY
KELVEILGDTNKPIEIEDLVKLRYMEAVIKETLRLYSPVPVTARNSKSDFMLPSG
LTVPKGCDVIIQIAGIHHNPRYWGADVEDFKPERFLKDEKPDAFVAFSNGPRN
CVGFCFFYNLLLRVSRGSESDRMKM

Table S9 Amino acid sequences of olfactory receptor (OR), gustatory receptor (GR), and odorant binding protein (OBP) in *H. assimilis*.

OR
>HaOR1
MFLMYYKKKPGMDIINNIDSDYLSYNNLPQKYKLIVNKNIDNSLFYSEKCWA

LTVFIGVLIFPLMATVSTVDSFLIKGESTKYMIHDLVIPFMDPEDRFKSPIFEIMF
AYTLYACMWYFVSFFGYDGFFGVCINHACLKMALYCQAFDDALKEVNEKAM
HKKIVEVIHEQNNLKRFVDLIQETFNFWLGVILIATITQTDRVYTRFIDTYKIITF
AMGVGMIYPNPKTNKRRIVCILMVLVSVMPFALMMLIDIYNSWKRRDILNILR
HTTIVGPFLGLFFKMFLMYYKKRPGMDIINKIDSDYLSYNNLRQNYKFIVNRSI
DNSLFYSEKCWGVSVFIAVLIFPFMATVSTVDSFLIKGESTKYMIHDLVIPFMD
PEDRFKSPIFEIMFTYTLYASLWYIFSFLGYDGFFGLCINHACLKMAVYCKAFE
DALKEVNEKTMHSKIVEVIQEQNDYTRFVDLIQDTFNIWLGLIVVATMIQIGTV
LYLISEGYGLDLRYIIFLAGTTLHIYIPCRYSAKLKYKASYIYFIISECVLLLGLL
ALTASCEFSRDSYANLLRGMGKCNLDEYSENCSNYDR
>HaOR2
MWLAFRKFGLDYDDFSTMIENVSILMIVLTINIYKNSTKRLEPMFESPNFEIATL
IFMLGICFGVVTLANVLAYIIVIVGYIESQMRALSEELRNIWDDSQHFYNNVKH
KVTDKINIMYYKEKIVDEFIKQSLRRIVKFHIANINLSHEVDQNFRPSLALEFSI
MAFAIIAELLGGLDKTYLEIPFTLSQIFMNCFIGQRLIDACCDFENSLYSFWFVFI
RCPQTGDYVSASVELSLAMCNGASILKFIYLKIYRKEVTNLIDQYLECHARVDI
KSRFYESLEKYLRGVKRRALMTWGALVLNGTIYISYGFLKPGRHLSEDLYVIY
GLEPMFESPNFEMAVVLMTISVVFGVITLANYRLLITVTIGYVEAQLLALSEDL
RKLWDDSETFYENYSEKELDIIKHVSPYDIKNVYIKHRLREIVKFHITGITLQHF
VENKFRFIYVLEFLFAAIGIVTELLGGLENTYLELPYSLNQVFLDCLIGQRLIDA
GNVFEDAIYDSQWENYNAENQRTVALMLENAQKTLTLSAGGLSPLSFMCLM
SVIRCTYSTYTALHSTVK
>HaOR3
MEVCKIYPKIKQICLMNSGREDCDIVDIYSEAVNVIKPKDRKTSIYVLPAIYQH
DLVGYCNIIINYRCPRSRDKVRVHIPFSFKAQASRRTSPLLKDYIGNNKFMKCE
SDDQNSLDNCSPTDCDLKYQGQRPFYEMNLQTCIEAPLCFADEDKELPNVVIV
PEINICKDLGVPLSIQDIYSLSTGLGTVTKILTKRDHVKLIVTIIRRNEMQAIFDG
INADYDKFNNLPEDYKEIVFDTIKKTKGLEKAWVIMVAITAGSYPGLAGICTIY
SSMFSDNPRRYMIHELALPFLTEEEKYESPYYELFAVYSIFIVWVIFVGFTGYDG
MFSVCILHVSLKIKIFSQNLKYLFVDTTDLSKIKRNLAEFVKDHCEVVRLIGEI
QKCFEVWLVGIFLNAVLQIGMALIQITSNNESDINAMYYLFALATVVHIYLPCY
LTSDVTHNAAEIANVAYSCSWELVQDKEIRKSIAIIISKAQNPIHFRALGMLTFN
MELFVSILQTSYSMYTLLRS
>HaOR4
MMTKTKTQGLVSDLMPNIKLMQLAGHFLFNYHSDNAGMTTLLRKVYSCVH
AILIIVNYVCMAVNMAKYSDEVNELTANTITVLFFAHTIIKLIFFAITSKNFYRTL
AVWNQSNSHPLFTESDARYHQLALTKMRRLLYFICGMTIFSVTCWVTITFFGD
SVRLLMDKETNETLTEPVPRLPLKAWYPFNAMSGTMYIVAFVFQIYWLLFAM
SIANLMDVMFCSWLIFACEQLQHLKAIMKPLMELSASLDTYRPNTAELFKVSS
SEKSEKIPDPIDMDIRGIYSTQQDFGMTLRGAGGRLQTFGQQNPNGLTQKQEM
LARSAIKYWVERHKHIVRLVSSIGDTYGTALLFHMLVSTITLTLLAYQATKING
LNVYAFSTIGYLCYTLGQVFHFCIFGNRLIEESSSVMEAAYSCQWYDGSEEAK
TFVQIVCQQCQKAMSISGAKFFTVSLDLFASVLGAVVTYFMVLVQLK
>HaOR5

MPVSVDIAPRRYFYVQYILLRFLGLGWWHHPDEGNTDNFPGLYIYYAIVTEIF
WVAGFVGLETIDPFIGEKDLDRFMFSLSFVITHDLTIIKLYIFFFKNQDIQEIVRIL
EIDLQQFYQNIEKNRRTIRITKILTASFIFFGWMTIGNTNVYGIVQDLRWRGEVA
LLNGSDKIPPRTLPQPIYIPWKYQSDVSYISTFLLETIGLLWTGHIVMTIDTFIGS
LLLHMSSQFAILREAITTAYDRTIIFLNNNISNNDDTVIEGFTNMGLGSYYERIV
RRYYTEEDIEKALESTLKNCFRQHQMLISCVEKFAKTYSYGFMTQLVSSMAAI
CVVMVQISQDASSFKSIRLVTSLAFFMVMIIQLALQCFTGNELTRQAGLVSEAV
MQCKWERMPPRLRRSLIITMMRAQRPLHLTAAGFAKMDNDCFLRVSVTFHR
KNVARILSLCTML
>HaOR6
MALRSQCGAFRKIVMHCVHGKPYLGKKWLRLTEDILRTNTSLNIRSLVSVLGP
GVAFKANRPNYNINSERNWLSSYDHTVGYSTYSTIVKSLLILLGCGELWSFFSS
NWSLDGITDGLNVILIQFGALYKYKVVMRHKKEFRELASSMESENFDLSTRR
RKMILEVWTKRNDSTLKLMLGLGTCTVIVWHIYPLMDDLDYNLMVAIRLPFD
YKTPTLYAVTYICTLIVFSYISYFVMANDLIVQAHLMHLLCQFAVLNDCFKNIL
RDCQSNFKDIDTRYLHLNQNFTKVYKNRLGKLVEQHQLILNNTMKLRNLMST
PMLIQLAVSTALICSIGFQIATSLNVNMTKGLMSLFYLGYNMFVLYILCRWCEE
IKIQSQKIGDALYSSGWENGIVMVPGVRTSILLILARANKPTSLSAGGMYELSL
EAYSNMVKTSYSALTVLLRLR
>HaOR7
MLFFPQPASASSVLLVHLGAVGALASGAALCGGGGTCACGALLHALHPLQLW
TVCGLHADRAAAIAAPLHYAAIVSAKKVVICVASGWIGLAALLAPLAAAQPP
LTYSIGLGSCAPDCGAGPGALGFCLIYTLLTLLLPTALVLLCSLKILRIARYHRH
RIAAAIYEVTLSAQVTVTHQRNPFSPPPPPRRRALSAVIQPLGSLAILYFPYYCV
LMWPAIAEPPQLLAAFSAVLLAAAPPVNGILYGIRSRAFKDSLRNYRRKRMTK
SEVTQEIQARTPSACGSRRPSLSAGSGCIRPLTTRRLSDAAAMGSRGSERPAQR
AASCNMLQDCQEVDTPKSRASAIPLIRAPPHVVLGRALGLEEGVNRERRRRQ
SPRIMVTRAMSDECESPSRRPLCRQHSRSSGALLGNMTYSPALLEKVNDNKA
DEQLLLSWPQRSQVTKNDVL
>HaOR8
MKLNKSKSTVNNEGVSSYTHFLEIPLKSVGCWDWYEKPKKLYEIFINNIYLCV
VLFVLLNVLLSLTVHLYTEWTDIMSSLDIIADGLPLLTSVLIVSYFAINKDELYS
LTKFMNANFKWHSARGLTNMTMMNSYKTAVNFSFFYTACTLFSVTMYVLMP
VIVHLWTKQPLQHWIYMDVTYTSYIVLIFLRQCLAQVFVALALGQLGVFFAC
NSILLCGQLDLLCCGLRNTRYTALLKGGVDHAALFNQYKGIEEDEKHNYLYN
KSEMIDSEYHYDDKVRNNFMGKKTQYDIYNKAYDKATSDALRECARVCQVI
NEYKDRFEKFVSPLLVLRVVQVTLYLCTLLYAATLKFDMITVEYLGAVALDIY
VYCHFGNQIILQADRVSTAAYQSAWPTMGVEPRRLLLNILLANLRPVVVRAG
RFLPMNLHTFVVVCLLM
>HaOR9
MLKFLRVTEEEYEKCAKGIINPELFYKNFYLLLKWFQVLDGPIPKWTTYAKIF
MTLCAITAQILLSLSIYHGVDNFDIPIMTEAGTYFIVMSYELLILSCTKLNIVDY
HKLQHSLKEDFLYVCNKGGKYREIFFYNQIETRKISMLAMIFIGNFGVCMIVTA
VSSLLYHLATHGPNEGKRPLLFPFWAFETDFGMTPTYEIAFIFSNICVTAYAFSY

IFMVVTQIVWIREIAIKADIVKLCIQDLMNGIHPTSDKERKNYFDFLIKYRMKE
IIQQHQSMYSLMGHYANVYKKLVLFEQKVSAPVVCLSAYSATQKLETGELNAI
LIILCIGAVTLLFIPSYLCTYLSIKVSSICYACWDIPFWNANRVIRPYLVLIMQRSL
RPLPLIAPGFDEVSVQTFSNKMASAYSFFNMLRQANI
>HaOR10
MIQNISKSFKRLEDPKYPLLGPNVTGLYWFGLWQCGNKYRDGFFNFIHFCSLL
FVISEFVELYFMRNDLMKVLFNISVTALSLVSISKTMFFIFYLPYWKTLIENISRE
EISGLQDKNLKVVDIMNQYKTYSRIITYSFWSVILLTNLVTILSPFLKYVTTETY
REMIKNGTEPYPQILSSWFPFDKTKFPGYLVAVAVHIIMTTQGAGVVAVYDSNA
VAIMSFLKGQMQILRYKCQLIFGDEKSIPKEDVLENIKEQYKTFNSIISPVMCV
YVLVCSIMICCSVVQLSLGEITVSQKLWVMEYTTALAVQLFLYCWHSNEIAYE
SLGVDQGVYSSNWWRADVQVRRQVLLLAGKLAPTFILDAGPFAKFSMSTFID
VSIFHNNYGLTIFEVITSYGRISRFVK
>HaOR11
MLKIFTCLEDPAHPLLGPTLWGLQKWGMWQPNKGISRIVYNVIHVLAIIFVVT
QYIELWHIRSNLEMALRNLSVTMLSTVCVVKAGTFIIWQEPWNFIFEYISSLER
DQLSNRSAVTKKIIKKYTGYSRTVTYFYWCLVTATVFTVIFAPLALYLSSFQRR
EEIKAGTEAYPEIMSSWVPFEKTRGFGYWSLVLVHTLICFYGGGIVANYDSNA
VVLMSFFTGQLELLKSDCERLFGDGIEFICYEEAVKRIRNCHQHHVELIRYSKV
LNSLLSPVMFLYIIICSLMICASAVQLTSDGTTRMQQVWIAEYLVALVAQLFLYC
WHSNQVLYMSLKVDEGVYASAWWSQCVRIRRSVALLGGQLNRPIMFSAGPF
TQLTVATFVGILKGSYSYYTLLSKK
>HaOR12
MRNYYILKGFCKRIFLVRSGNFWYEEGVMGDDNCISYKISKYILFSVYGFVTL
LEIMAALIGDFPEDEKRDSVTFAVSHTIVMIKIFSVISNKGLIKKLIYDLIKVCEI
HEEEVLMKEKYRIMKINVLAYFITVYGSAACFVFEGLRKMFEGSHFVTVVTY
YPNFEDNSVIATVVRIFTTIVLFVMMLTMIVSVDCFTVIHIIMLKYKFITLRNYF
EKLSDGFEHEIKIKNSQISATKLTKGLIEGIKMHKELLRLTKEIDKAFGTVMAL
QLCQSSGSAVSLLLQIALSDQLTFVAGMKIFFFVVALFFLLGLFLCNAGEITYQA
SLLSDSIFYCGWHLCPSQHSQQRNLGRLVLLACAQAQRPLVMKAFKMLELTY
GTFLLILERGLLERIAA
>HaOR13
MPEKKFKSFNETFPHCAFALAIALLYPNRANLNKRKILFAIVIVVNSLILFWFLL
YLVKCAFMLDIYNLSRNVTIGILASLFFFKTFYVHSKTDKFAELLKKITDDML
MANDMEKEYQEIFEYYIKIGKLGQTCWIIIPILLSSQFPIYAGACMIYENLKSDV
GKKYMVHEMELKFLEDKQYDTPYFELIFAYNLIQCVVLSLNFAGFDGSFCIAT
NHLRLKLKLLAHKVCKAFKIAKSRHELESMVKEAIRDHQEALIFHKDLQEIYG
GWLLMVFLLSSLLISLNLFQLYLSQRIDPKYTIFAISGVIHMFTPCYFASNLMKT
SEELSWDLYSAAWEKWADPAVTKLLIFMIAKSQQTLILTGKGMVYFNMQLFIS
VLQTSYSFFTLISS
>HaOR14
MSFLKSITYDIVNKDLFEFNIKYLTLVGLWTNKDWSTNKLRLYKLYEVVLHILS
FVFIIVTGIGTYQHKENIPIFTSSLGKCLAAYNFVSKIFFFVMKRKQLSNLIHEIR
SSGDEVSVGKRQLMVIHVIMITTISTVLATAFSILSLLKGEMTIEAWMPFDPMK

NRMSLLLAAQILVVYFVVPCVYRAYAIQGIVCSIVMYFCDQLVELQQRLKNLS
YSKERERIMREEFKEIVKKHIRIMRYSNILKNIFKEFFLIQNLAVTMELCLNALM
VTVIRLEEKTLLASFLGYLGLALMNAYIFCYLGNELIIQSTGLAQAAYEASWTS
WPVDLQKDLLLVIRVAQKPLTLSAGGITNMCIKTYSEALYNAYSIFAVLSDFVD
>HaOR15
MSTKYFDKSIEKVQFIFLCSGLTLQSKKLTTIKKLKSRLFYLINFFWLNSDVLG
AIFWFIEGIKNGRTFVELTYVAPCITFSFMANLKAIYLIHYEDKISDLIDKLRDLE
ASDVIIDKSRDDIRKTDSIFLHLVLKCSNILNIVLLVTFIVSPFILIAIKYVQTNKV
DLILPFLIVYPFDSHDIKYWPFVYVHQFWSVSIVLANICGADYFLYICCTYLRT
QFRLLQHDFTNIIKKNRHLSDFDLGRFDLEFIKLVKWHQELIRLSDILELINAKP
TLFNFVSSSILICLTGFNVIAIENVAFAVTFLVFLTASLLQIYLLCFFGDLLMKSS
MEVSDAVYNCKWYNLNAKSRKNLIIVLTR
>HaOR16
MTDDSREESFDLDFENVFGVMTSAMRMNRSHPDIKRNIKWVFQFGVMHGVF
SLMFGGLVYSIIFHDLKNNDYTQACANGILCVIFNVVTFSYILMLWHQNIFKD
MIAKINRDYELAKHFTKDEQNIIKTFAGKGQRVIKLWLVVGILSAGLFPIKAIV
LMIYYTTVSEFKYVHLFDLTYPDSVEEVKNTLGVFIVLYIYFLYCDLYSTTIYIS
FVPLGPVFMLHACGQVELAKIRVMKVFSENLTIEEKNKRLSDIAKLLDNIYSFV
DKIKTSFKMLYELTLKGTIVIIPISLFQILEGEELHFAVYSCNWEKQWNKKNRSL
VILLLQRTSQPVAIRTLFRDLCLDALTDVRQ
>HaOR17
MNYYFGLLRSAAWRCVAAHTTLLQGAGYMRWRGADKSTSRVHFIYRKIVFAI
TSLYLLQECIYAYRERNDMTKLSKVMFLLLCHITSIAKQLLKCYVSFQDPIYNA
ANPKAQTLLQSTSRGARLLLSAYTGCAVLTCTLWLVFSIMHRVDGHYVEFPF
WTGFNTDPPIVFVVVLIYSFYVTTLVGIANTTMDALMATILYQCKTQMRILRN
DFENLPERAKVICNETGEGYERVLMMLLVRGFQHYRRVIGTAKSLQDIFGVAI
LVQFGIGGWILCIAAYKLVSSERIVCSVYSMQWLKTPPHFKRTLVLLMQFVRR
PLRPAVGRIIPLSLDTFVKILKSSYTFYAVLRQTK
>HaOR18
MRILKSEDLYLNRAKFVMKYLGVWVPRINENKIHKSYRMFMMSLQYLFLIFQ
IVYIVQVWGDLEAVSQSSYLLFTQACLCFKVTVFQVNIDKLKDLLKRMNDGIF
LPQSTEHERILKNQAKRIKRLLLAFMISSQTTCGLWALKPLFDDAGSRKFPFD
MWMPVRPELSPQYELGYAFQLLTICMSAYMYFGVDSVVLSMVIFACAQLEIIK
DKIMNSRNDDNETIDRKNILTENNNKLIECIKQHQAISENLHTVLYNCAWYEQ
DANFKRMICFAMMRMSRPIVLRAGHYISLSRQTFVSILRMSYSYFAVLNQTNL
N
>HaOR19
MIFLENLAAAFGNFPKVEKNSAIMFSAIHNIILVKMFLLFYYKSSIKRINYEMA
SLMKDIEDGDTMIMQKKKVLWGIVFYAITVYLSLIAYGVESLRKFLVEGTPFY
TVVTYLPDYYDVSLVASGFRVFFYLTWLYMMLPMMAADCMPIIHLIIIAYKFIT
LCKHFETIKMEFDRNLLIMSNKKATELLKTGCIKGIKIHQKLIFLAEEIQRIFGVI
MSLQVCESSAVAVLLLLRLAASLLANSIFFCGWHLCAMDKQSHKDIRRIVLVG
CAQAQKPLILKSFGVQDLSYSTFVSVARMTYSVFAVFYQRRD
>HaOR20

MDESSRNKAKLEINESLTLSIFSMRRIGLSFDKPKTSSAYFRQKILFVVSVCGIC
CHVFSEFINIILTFASSPRVEDVVPLFHTFGYGALSIAKVFVLWYKNTVFGELID
ELAGIWPMPPLQEDALVIKEKSLTALRISHRWYFCVNVMGVWFYNLTPIIIYFY
RIWQGRDAEIGYVWESWYPFDKHQPIAHVAVYLFEMFGGVTCVWIMVSSDL
LFSGMASHIALLLRILQRRLETLGTPEQSDEENYEEIRSNIKLHQRLIRSQKPIAF
TAMKFTNISLVTYSSILTRSYSYFALLYTMYNDS
>HaOR21
MTNLIDKYLECDARVDAESRFAKNLKKKLKMVKRRAMLTWGALALNGVVY
ITLPFLKPGRHLTEDLYVTYGLEPMFESPNFEIATVLMTLAVVFGVFTLANYRL
LITVTIGYVEAQLLALSDDLQKLWEDSETFYEKFSKKELDIIKHVSPYDIKNVY
IKHRLREIVKFHITGITLQHFVENKFRFIYVIEFLFAALGIVTELLGGLENTYLEL
PYSLNQVFLDCLIGQRLIDAGNVFENAIYDSQWENYNAKNQRTVALMLQNAQ
KTLTLSAGGLSPLSFMCLMSVIRCTYSTYTALHSTVK
>HaOR22
MDIINEIDNDYSSYNNLPQDYKLIVNKHIDNSLLYSEKSWAITVFITVMIFPFMA
TVSNVKSVLFDSEPTRYMIHDLVIPYTDPEDRFKSPIFEIVFMYMLYACFWYVL
NFLGYDGFFGLCINHACLKMALYCKAFDEALKEVNEKAIHKKIVEVIQEQNN
FKRFMDMIQDTFNIWLGLILVATLTQMGTVMYLISEGYGLDLRYIIFLAGTTLH
IYVPCRYSAKLKHKVGNAPACPLVLQMFLGGDDRLTSVSRDSYAYLLRGMGK
RNFEEYQENCTNYDR
>HaOR23
MATTYSTAFMFTVCTALISYGFDGLVEVIHADGTFTTVITAWPSPLDKSVLANF
VRIINFLVWWAFVIRVSATYVVIITVATALSHQYKNLQSYFYKLNDLFEDAVDD
RTQNENEIKYMKALEVGIKLHSDTLWCKTQFQEICSPVFSGSIMINIFVLCMLM
LQMVNSERTLLNGISTLTTSAAVLLSTGCIMWNAGDVTVEAAVLPSAMYSSG
WENCQDKVSRRIRNLLVVAMQQGQKEVIIRGFGIFEISYQSHLAIVKSSYSTFS
LLY
>HaOR24
MSRLLMIYHAAVGICGVLFTAYPIINKALGDEVYLTGYIPFDTNASPVFEVAAT
YMGILITLQAYGHVTMDCTIVAFYAYAKVQLQILRYNLEHLVDETEVRRPRLK
YVDHDAEAKNILHERFVNCIKHYEQIVWYAKEIECIFSEAMVLQLFVVAWVIC
MTMYKIVGLPLFSAEFISMAMYLGCMLAQLFIYCYYGTYLKFESDLINESIYK
SNWLLMSPGFRRHLIVLMERGRRTIEPCTARVIPLSLDTYISVLRSSYALFTILD
RK
>HaOR25
MTSTVRNFLTKLEDPERPFLGPNVKWLELWGLLLPKTKLKKFMYMMIHTLM
FIFILTEFVEIYLKRSDVTVILQNVKYAMVSVVNFNKVVTFIVWQKYWKSIIDF
VTVTDMERRKSQDKTYQDIIKKFTRYCRSITYWYWFLTYCTVVMTISQPLVK
YLIILYNGNDSDTIPEIVNSWLPVDKHTLPGYLLLMSYQIYAAFYSGGWMTSF
DTNAFSIMIFFQCEIELLRKDGQFLFGTLDNPRSKEEAKMRFAFCSKRHNDLLR
>HaOR26
MTGTAFLLFTNLSHATKILNILARRDDIQAIIDNADLVLRAERSDEGRDIVKRYP
YDTSKSPAYELTYIHQVIAISVAAFLNLNKDTLVTTLIAQCRCRLRLVGLALRN
LCNDSYSIGDTSANIDLTPSPKTLILTPAQEEQVRIRLRGCVQQHQKALAAAVH

LQKCFSEPTFAQFTVSLVIICVTAFQLVSSEDVAGAAYDFPWYACSVRIRKSILIL
MRRCRRTTKLTAGGFTTLSLASFMAIIKTSYSMFTLLQQVNERN
>HaOR27
MYRKLASAMESPYFDNSTPKRKALVKFWSQRNERFLKLLLILGSCTLGAWHI
YPMVDDIDYNLMVSARFPFDYKTPNRFPIFYIIVLVVFNYGSLLVMINDLMMQ
AHLMYLLCQYTVLANCFEDIINDCLGDENKLNEKHLIMTEQFKRRYLERLNG
LVEQHKFILNNTMELKQSLSVPMLAQLAASAMLICFVGYQASRSENIGNSVY
CSGWERGLTAIPGVSASLLIVAIRARKPIVLTAGGLFDLSLASYTTVIMYL
>HaOR28
MYTYFKIVVFWLNKKKILNLLKFLHCDEFKPEEREHIEILRKSIKTSRFVMTYY
STMFGIVLPLTENFEILPTNVEYPYFDVYTNPTYAIVYLHHVLGQIEILAYNLRN
FENMAERKRKRDLRKNHGIEDPRSHPIQIIWMAIYLTCMLIEVFILCWFGNELI
LKSLELRRAAFEGPWLKTDPKTTMFIVIFLERCQRPLRVTAGKIFTLSLDTYTY
LINWSYKAFAVMRNTKK
>HaOR29
MKADDFTTPYGLEPLTNAPKREICLLILFTQECTIMTVVLNYQALLLFLIAHTA
AMYEMLSTEMLAFDKYEDTSESKLLVKKRLPLLIRRHTLTLNIIKNLKALYSM
PIGVNFGSNAVCMCLFFYLPLRECVTFSPVLIYCFLVFFLYCFLCQRLINASEFF
ENAVYSCGWEKFDVKEQKTVYVMLLQAQQPITLLAADIIPVNIYTFATTLQAI
FKFITVVKF
>HaOR30
MTLVVFNVWGHLKILRYHLEHFPKHESSVNPAVYNEKVLLLLKENIEHHKLIT
EFMSLASEAFGPVMCFYYLFNQVSGCILLLEISTLEMAAFASYGTLTFIVFQQLI
QISVIFELIGSQSEKLKDAVYNLHWEHMNQKNKTIVLFFLYKSQTPITLKAMG
MVPVGVQTMSAIIKTSFSYFLMLRTVAEN
>HaOR31
MNTKLSYRSVVPHLFRLRLSGYYQIDPKSPKIKRILHSIYMRFTLIWIVVYTTQ
QAIKLFEVQDDIDKVMATLFLFLTHTDSIYKQMILWIKSDEIEELLDIMRGPLY
NQEDSDHMEYLTDVARYALLILRIDNILALFTCFLWVILPFVLHLQGKPVEFAI
WLPFDVNFEPA
>HaOR32
MNENFLKPYSFICVLDFTTVDALYYIMTTHICSHFTILSNEIQHLDEKTSYRLK
DIVKKHQYILKLSQDLEEIFCVPNLFNVLVGSLEICALGFTLTMGELAQIPGVV
LFLLSVFIQILMISVFGEHLIQESRKIGEAAFKSKWYNMDQKSKKTILLLMLR
>HaOR33
MYHKEKIVNEFTKQRLKHIVKFHIANINLSHEVDQNFRPSLALEFSIMAFAIIAE
LLGGLDKTYLQFPFTLVQIFMNCFIGQRLIDACDDFENALYSCKWENFNTTNQ
KTVYLMLIMSQKTLTLSAGGVTKLDFNCLMNVLKSAFSVYTTLKSALK
OBP
>HaOBP1
MTKSIVEMSLNKTIAALFLIFTSYCYALTCHSQRGGKENEFKRVINTCLRQMEN
NNSRNSNEDWSDWDNYNQRNQRERDNRHENRENKMGNRDIEKGERDRNK
NKRDGANERHNRQQNRDGVNENISNRMDDGYNSMGARNVNVNMNGPDSG
SGRSRNDMTKNYGKNDQINGRDEFFQSEEFDGDNPTVQQYNYHPSPQPSRRY

RREKRVEMNSGQRSQYNPHSQKSNSHEGNNEKRNSSNSSSNDADRACVLHC
FMEKLHMTGDSGMPDRYLVTHAFTKDEKDEDLRDFIQESIEECFQILNNENTD
DKCEFSKNLLTCLLEKGRANCDDWNESTSLLFE
>HaOBP2
MQCSDMSDLLEFNETTFSSVSSRLPARGSMLVQVVLATLLALGAAPCVLQRA
CPPRAPRPPSSVCMHRLHPDDNVVVAPPGQDARAHKARRGAERARVLLPALL
RLSLAAVLYLLPLRVSQGGEMIAIFVFVMFFGIREIAADCKNCGMLGKDEKAM
FRAHSEACRSHSQVDPKLIESLLNGELVDDPGLRKHVYCVLLKCKVISKDGKL
QKAAVLGKMAARGDGKNVTKVLENCANQPGESPEDLAWNLFRCGYDKKAV
LFGHTRAAPAGNDA
>HaOBP3
MNSNCFILIACAIFSVCNSSYVDKLLKCSVNDEECFKKLLNKFLEDNSDNGIPE
INIPPLDPFELKNVVVTVPDLINATFIDGTVSGAKKCVINSFKIQSDKGFTKVGL
TCDLVLKGTYSLEGTGPLLKNLLGGDFLRGSGNMELKLNKIKMNLGVVYHFI
KKDDGEVYYKCHRDKFQYDCDVGNAHLTSEKIFLGKEDATELVVGFFNQNW
KMLEKSFGKHLFDTALNIFNDVLQKFLESTSVKHFIIEDLSNLVKN
>HaOBP4
MYKSNLSYLFLVPSLPKCHVHDEDCLKSLAQKAITDISNIGVPEMGIRPIDPFQ
LSNVTVSIFDIINITLVDGEAKGIKDCVVDSLVSQVEEGKTKVAYTCDLIIEGKF
NLQAASPLFQNLLGGEKIHGNGNAKVELDKVHMSLEFSFHYIRKEDGNIYTK
FHPEEYEYDYSIGDMRISADNIFLGDKDVSKMIVVWLNENWKYAMQTFGRSF
VDVAMGLFYDILQQIFYATPTKSYIIEDLSSLVKHEE
>HaOBP5
MHYTLINSPANGRLHAQQLARCLESDPSLTDPSSGPGYRRELYCFIKDFHLPEA
RKAIARAFIADCVKESGAKIEILAEARKGKFANDEGLKEFTLCFFNKAGILTPD
AELKVDVALAMLPAGVDKNVPLSGKQKEKAKIYIEQCAKESGATSEILSEAKK
GKFADDEGLKKFILCFFQKAGIISPEGELNVDVALTKLPVGIEKDFTGKILNECK
NRKGSTKADTAFEMYKCYYYNSKQRILYD
>HaOBP6
MARVIVIFFLLPTLAICFREGNIHLLEEEIQAALNSCTQTNHSKNQNDNGKRQK
RFDSNSYPITRIDANPKEEVNPYNHIRRNTSLIDQMNVLNGTDYDYPGYRAGT
GGEKYVKSIPRPALGKDIKNNSINYNVNDRIKRNEPLINNHDNDQCLSQCVFA
NLHVVDTQGIPREPELWNRIRTSVTSLQSRVLLRDQIRACFQELQSESEDNGCS
YSNKLERCLMLRFSDRKINGTQTATS
>HaOBP7
MMRKTCQPKNNVEDDKIDQLIKGVFIEEKEVMCYIACIMKMANAIKNGKLN
YEAAMKQADLLLPEEIKEPAKEAITAERSDADVKKWFIQQALECTKEHPLTGE
EIQMLKEHKIPDQMSAKCLVACLFKRIDWIDDKGTFNKENAYKLSEREYPGET
EKLENAKKLYELCSKVNEEKITGDNEVCERSVLIAACLTQHANTMGFLI
>HaOBP8
MYRAIVFLFAIIVACQANVAVTPPIKCGPLPNMVLWCIDTPTVIKAEVATRCSSA
VSECEKTTCTFRESGWLDGDKLNKEKLSAHFDKVGEDHPEWQAAVESLKTS
CLRINLPAQGVYLNCPAYDAAFCIYTSFIKNAQSSVWKTSPRCDYPRQFAAAC
PVCPEDCFAPLVPIGSCNACLTLPRSP

>HaOBP9
MFRKVSALLCLCVFSISLSDSAISAESETRCRNPPTAPQKIERVISLCQDEIKVSI
LREALDVIKEEHTMPAERRRNKREVPFTHDEKRIAGCLLQCVYRKVKAVDGY
GFPTLEGLVGLYSDGVNERGYFMAVLEASRECLMRNHDNFSRSVPMDNGRN
CDVSFNIFECISDRIGEYCGNSGL
>HaOBP10
MGVSRDGRQQGSARRWLVALLACNVMVVRGNVDVMKDVTLGFGEALQHC
REESQLSEDKMEEFYYFWNDDFKFEDRELGCAIKCMGNYFNLLTDSHRMHH
ENTDKFIKSFPNGEVLSKLMVELIHKCEQKHDSEPDHCWRILRIAECFKASCQ
TEGIAPSIEMLMAEFIMESEAM
>HaOBP11
MLRIVSCLFIVLQVVSSQPPPQCRRPPPGIKNPGECCKIEPIFKEEDFKDCGIEKP
DDAKNFKPGPPDCSNSICLLKKYNLMKDDSDLDTDEAAKFLDKWVESNKDF
KDTVDKAKDKCLKDDLPGPPEICTPQKVVFCVAHTIFTECPKWEDTDNCKKL
KDHIEECKSYFP
>HaOBP12
MASFSYLIVFVVVLAAVPRSVKSTAEVMSHVTAHFGKSLEECREESGLTPEVL
QEFQNFWSEDFEVVHRELGCALICMSNKFSLMEDDARMHHVNMHDYVKSFP
QGELLSAKMVELIHNCEKQYDDITDDCSRVVKVAACFKENCKKEGIAPEVAM
IEAVLEQY
>HaOBP13
MKIQLAYAVIYLAISVNSYKHKFFSQNLDSEPSLSIQYARDKRSDVITNECLME
MYPRNLYKYPLHIDRNDIPCIIHCVLKKFGIMTNDGIINIRNYYRRVQAIHRYDP
RILISDVGETCAQNINGMNLDHDVCKKAKVFNDCTQLYVISFRDTDE
>HaOBP14
MTSFSLVFLVVAAAINLGTVHAISDEERNNIHLELLPILAECSKEYGVTDEEIKT
AKESGAIDGINPCLMACVFKKMNVMNVKGLFDVDKAEEITKKFLPNGDDQN
KATDIIKSCASVNDKDVSDGDEGCDRAKLLFECFIPFKGEFIKSS
>HaOBP15
MLKLFLTLLLYGVILNFRVSADSTEDLKQKYVEMIMGCAKDYPVTADDIKQL
KNKQMPDSAKCLFACAYKTSGMMDDEGNLSVEGVNEIAKKYLSDDPERLQK
AKDFTDVCKEVNDIKVSDGTKGCERAALIFKCTIEKAPQFDFDV
>HaOBP16
MMNTEALSTTESAFSMEENNKTDLDIMAIMLDCNDTFRVEMPYLDSLNKSGS
FPDETDRTPKCFIRCVLEKSEAASEDGQFNVTRTAELFTQIRNVPQEDLTEMAT
PCSERSESCKCERSYQYLKCIMENVINKYDTP
>HaOBP17
MDTFAMTVDQKAMIHKHFEELGIECIKDNIMTNDDIRDLRSRKLPTGENVPCF
LACMFKKLGIMDETCFLQKETALDLAKKVFNDEDELKLIEDYLHSCSYINSES
VGDGDKGCERAMMAYKCMVENAEQFGLDI
>HaOBP18
MEGYLALCSVLICLIGKTRALDGEMAELAKMLRDSCADETGVDTGLIDKVN
AGADLMGDAKLKCYIKCVMETAGMMSEGQVDVEAVIAVLPEELQKHADNM
RACGTQKGSDDCDTAFLTQSCWQKANKADYILI

>HaOBP19
MKTILVLAICFVAAQALTDEQKDKLKKHRSECLAESKADEQLVNKLKTGDFK
TENEPLKKYALCMLIKSELMTKDGKFKKDVALAKVPNAADKPAVEKLIDTCL
ANKGNTPHQTAWNYSKCYHEKDPKHSIFQ
>HaOBP20
MECGKDHPVPVADMLELHKLVVPKKREVKCLLACTYKKLGTMNAKGLYDL
EAGYKIAEKARNGPGDDKRVENGKKIADICNKVNDEPVTDGEEGCDRAALVF
KCFVDNAFKLGFKLQM
GR
>HaGR1
METNSLNTRTHGFNKRNEVRKRIMFEQGDGKDMKGYEAKDVYGPQITENDG
ELLDKHDSFYLTTKSLLVLFQIMGVMPIMRVPRDAKTTKRTTFNWISKATLWA
YLVWSFESIIVIKVGRERLANFQQNSNKRFDEVIYNIIFLSILIPHFLLPIASWRH
GPEVAIFKNMWTHYQLKYLKITGTPIVFPNLYSLTWGLCIFSWALSFAVILSQN
YLQDDFELWQSFAYYHIIAMLDGFCSLWYINCNAFGTASRGLATNLHKALQA
ENPALKLAQYRHLWVDLSHMMQQLGRAYSNMYGIYCMVIFFTTTISLYGALS
EILERGLSYKEMGLFVIVGYCMTLLFIICNEAYHATRKVGLEFQVRLLNVNLG
GIDRSTQREVEMFLVAIAKNPPIMNLDGFTNINRELFTANISFMSTYLIVLMQFK
LTLLRQGTKVLKKISRAIFNITTAATDDVEYEE
>HaGR2
MIPDHLFEEGINNSLFRNEMKHVQLSKVVYEKTQKDYEREQRNLLSSQDGDI
CEIHDQFYRDHKLLLVLFRALAVMPITRSRPGTITFSWRSRATIYAIFFYIVATVI
VLIVGYERITILRSTKKFDDYIYSILFVAFLVPHFWIPFVGWGVAHQVAIYKTN
WGKFQVRYYRVTGENLKFPNLKTTIVIISVGCLLLAICFLLSLCALLDGFLLRH
TIAYYHVITMINMNCALWYINCKGIKIASQSLSECFRRDVHLECTSSLISSYRFL
WLNLSELLQSLGNAYARTYSTYCLFMFFNITIAVYGALSEIVDHGFGFSFKEM
GLIVDALYCCTLLFIFADCSHKSTLKVAAGVQDTLLSIDILSVDRPTQKEIDHFI
QAIEMNPAVVSLKGYAYVNRELLTSAISMIAIYLIVLLQFKISLPKDLST
>HaGR3
MGVLPLTRSSSGLNQFHIASPSMLYSVICYVCLVGYVIYLSIDNVQILRTAEGKF
EEAVIEYLFTVYLFPMLVVPIMWYETRKIAGILNGWVDFEICYKKLSGRILPVK
LYKKALAIAVVIPMLSTSSVIITHVTMVHFKLMQILPYIFLEILTYILGGYWYLL
CEILSVCASIVAEDFQQALRHVGPAGKVAEYRALWLRLSKLARDTGIANCYTF
TFVNLYLFLIITLSIYGLLSQISEGFGTKDIGLAVTACCSILLLFFICDEAHYASHN
VRTNFQKKLLMVELSWMNTDAQTEVNMFLRATEMNPSQISLGGFFDVNRHL
FKSLLATMVTYLVVLLQFQISIPDDSQREMDTDENQKQLNDTTVTEATTITSTL
ATTIMTTLAKRKKKQ
>HaGR4
MPGSENVKTRQDECRVQGPLALALRVLRILGAAPLNMTRIGQTYVVTVSKKA
AIYGCILTLMTLWIEIMLAVFYIEIYKMSLLELENALFLFEIVQSLKAMREKFHL
LVDVVNSDSIKVDVALSTYKSVTTFTISYLSICELVREVNLRSGFYMYLLFSSSII
YLMLTVCNLLDSLHLYSMSYPGRFLLTSVCLQTMWILWHLKNAVQFVEPCHK
ITHEVNIIRCLLAKLMYNMTPVGKRVSLELDLMFKQLTLNEPTLTPFRMFAVQ
RSLLTTVFTSIICPIFFIVSFLASLGMLIP

>HaGR5
MMTLNRFTFGPHAEVHDKEAVLCSETLTGYDLAVGYFADVVSPSEQDHVAKV
LFAKKDGEESDKLTSLSKEVTLDVTSLHKLYDLLHQCSEQVNSVMSLSMIVM
LLTSGLSTILLLKNTIVVIKNISDMYNPSKVRSLIAYVFGRSLKYIILLAVPCYIA
NNTKGHVITIRSMIHDSLNGIQLGQPLCGINFRRRSFRTDTTWEPSREEPILSLK
VGNAPACLLVLQMFMSGGDHLTSDPTMVFRKKSVPLTMELAL
>HaGR6
MKLTIIVGQIFGLNPVIGVTDVDSSKLRLLKGYEHAVYFTYSFAFLVLRSLAVSL
VASQVHTASRQPVYALYDVPSAVYCIEVQRFIEQIHGETVALTGLQFFKIKRRIV
LAMAGTIVTYELVLLQFTGITPTVAPLHDDIEYKA
